# Differential proteomic analysis of the secretome of *Irpex lacteus* and other white-rot fungi during wheat straw pretreatment

**DOI:** 10.1186/1754-6834-6-115

**Published:** 2013-08-10

**Authors:** Davinia Salvachúa, Angel T Martínez, Ming Tien, María F López-Lucendo, Francisco García, Vivian de los Ríos, María Jesús Martínez, Alicia Prieto

**Affiliations:** 1 Centro de Investigaciones Biológicas, CSIC, Ramiro de Maeztu 9, E-28040 Madrid, Spain; 2 Department of Biochemistry and Molecular Biology, Pennsylvania State University, University Park PA, 16802 USA

**Keywords:** Enzymatic hydrolysis, Bioethanol, DyP, *Pleurotus ostreatus*, *Phanerochaete chrysosporium*, Lignocellulose, Extracellular enzymes

## Abstract

**Background:**

Identifying new high-performance enzymes or enzyme complexes to enhance biomass degradation is the key for the development of cost-effective processes for ethanol production. *Irpex lacteus* is an efficient microorganism for wheat straw pretreatment, yielding easily hydrolysable products with high sugar content. Thus, this fungus was selected to investigate the enzymatic system involved in lignocellulose decay, and its secretome was compared to those from *Phanerochaete chrysosporium* and *Pleurotus ostreatus* which produced different degradation patterns when growing on wheat straw. Extracellular enzymes were analyzed through 2D-PAGE, nanoLC/MS-MS, and homology searches against public databases.

**Results:**

In wheat straw, *I. lacteus* secreted proteases, dye-decolorizing and manganese-oxidizing peroxidases, and H_2_O_2_ producing-enzymes but also a battery of cellulases and xylanases, excluding those implicated in cellulose and hemicellulose degradation to their monosaccharides, making these sugars poorly available for fungal consumption. In contrast, a significant increase of β-glucosidase production was observed when *I. lacteus* grew in liquid cultures. *P. chrysosporium* secreted more enzymes implicated in the total hydrolysis of the polysaccharides and *P. ostreatus* produced, in proportion, more oxidoreductases.

**Conclusion:**

The protein pattern secreted during I. *lacteus* growth in wheat straw plus the differences observed among the different secretomes, justify the fitness of *I. lacteus* for biopretreatment processes in 2G-ethanol production. Furthermore, all these data give insight into the biological degradation of lignocellulose and suggest new enzyme mixtures interesting for its efficient hydrolysis.

## Background

In the fungal kingdom, white-rot fungi (phylum Basidiomycota) are the only microorganisms known to be able to alter all plant components, including lignin, cellulose, and hemicellulose [1]. The applicability of this potential for a number of biotechnological processes, for instance, as a tool for lignocellulose pretreatment in second-generation ethanol production processes, has been suggested [2]. However, for this purpose, the degrading microorganisms should display some desirable features, such as consuming low sugar for its own growth and promoting a high lignocellulose deconstruction, to render more accessible polysaccharides for enzymatic hydrolysis and thus increase fermentable sugar yields. To date, considering these fundamentals, very few fungi have been shown to be adequate for biological pretreatment in this type of processes [3].

The white-rot fungus *I. lacteus* can degrade different lignocellulosic substrates (e.g. corn stalks/stover or wheat straw) yielding high sugar recoveries compared to other fungal treatments [3-6]. Furthermore, a positive effect on glucose yields from lignocellulosic substrates has been reported when Mn^2+^ was added in *I. lacteus* cultures [7,8]. This extraordinary capacity is mainly the result of a high metabolic versatility and secretory potential. While different sets of hydrolytic enzymes are implicated in this process, the pool of proteins secreted by *I. lacteus* during the biopretreatment of a lignocellulosic substrate remains unknown.

Secretomic analysis, apart from being an excellent method to understand the biological mechanisms of lignocellulose degradation, is a valuable tool in the search for new enzymes or interesting enzyme complexes in the biofuels field [9,10]. For this reason, publications documenting fungal secretomes have increased in recent years. Most of them have been performed with ascomycetes and are focused on enhancing the enzymatic hydrolysis of lignocelluloses more than on the pretreatment step [11,12]. Among the few reports concerning basidiomycetes, nearly all have dealt with the secretome of *P. chrysosporium* grown under several culture conditions [13-15], since the genome of this organism is available from 2004 [16]. However, due to the rapid growth of genome sequencing and the associated ability to perform protein homology searches, the secretome database of basidiomycetes is currently enlarging. To cite some examples, the secretomes from *Pleurotus sapidus* growing in submerged cultures either on peanut shells or on glass wool [17], *Phanerochaete carnosa* on spruce [18], *Ganoderma lucidum* on sugarcane bagasse [19], and *Trametes trogii* on *Populus* wood [20] have been reported.

The aims of the current work are to get a deeper understanding on the dynamics of wheat straw degradation by *I. lacteus* over the time and to search for interesting enzymes and/or enzyme complexes for biopretreatment and enzymatic hydrolysis processes. In addition, the *I. lacteus* secretome’s composition after 21 days of solid state fermentation (SSF) on wheat straw, in the presence and absence of Mn^2+^, will be compared to that released either in liquid cultures of the same fungus or in SSF cultures of two white-rot fungi, *P. chrysosporium* and *P. ostreatus,* grown on the same substrate. The latter fungi cause different wheat straw degradation patterns when cultured under SSF conditions [3] and offer the additional advantage of having their genome sequences available. Advanced proteomic technologies, such as high-throughput nano-high performance liquid chromatography-tandem mass spectrometry (nanoLC-MS/MS), have been used to provide information on the physiology, diversity, enzyme interactions, and even kinetics of the expression profiles over the time, either from whole secretomes and from proteins isolated in two dimensional (2D)-gels.

## Results and discussion

The most significant hits from the proteins isolated from the 2D-gels, in terms of score and sequence coverage from both databases, are gathered in Additional file 1: Table S1. Protein identities provided on the basis of a single matching peptide, were considered as tentative. The functional classifications of the proteins identified from the extracellular pool of proteins (EPP) analyses, from JGI and Uniprot databases, are collected in Additional file 1: Tables S2-S9.

Before discussing the experimental results, some general considerations should be laid down. In the case of 2D-gels, MS/MS analyses showed that a protein can be identified in several independent spots. In some cases this observation may be the result of the coexistence of different isoforms or closely related gene products [21], but the presence of protein fragments from proteolytic cleavage cannot be ruled out. In fact, some extracellular proteases, which may have digested susceptible proteins either in cultures or during sample preparation, have been identified in the present work. In addition, some spots contain more than one molecular species. A probable cause is co-migration of protein fragments with other full length proteins. It is also possibly due to streaking of proteins observed in certain areas of the gels, presumably because of the impossibility of getting completely rid of a pigmented material contained in the extracts produced under SSF conditions. Additionally, it is worth mention that the correlation of the predicted molecular mass (MM) and/or pI of the hits with the values deduced from gels for each spot is not always accurate. This can be due to (1) a differential pattern of post-translational modifications, such as glycosylation, and (2) a match with a homologous protein from a different species.

### Secretome of *I. lacteus* growing on wheat straw

#### Comparative analysis of the proteins secreted over the time

**Figure 1:**
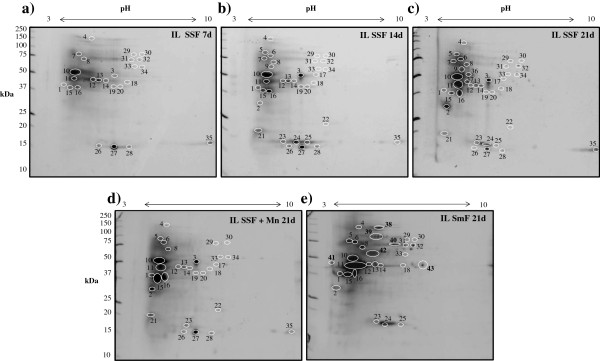
**2D-gels from *I. lacteus *secretomes released under SSF and SmF conditions. (a-c)** 7, 14 and 21-d wheat straw SSF cultures, respectively; **(d)** 21-d wheat straw SSF cultures with Mn^+2^ supplementation; and **(e)** 21-d SmF cultures in CSS medium. The spots analyzed are numbered on the gels and the proteins identified detailed in Table 2.

*I. lacteus* degrades simultaneously all components of wheat straw (Table 1). The biopretreated product keeps high sugar concentration with improved accessibility for further enzymatic hydrolysis aimed to second-generation ethanol production [3]. In order to study the major enzymes involved in the degradation of wheat straw and to investigate their variations over the time, the secretome of *I. lacteus* after 7, 14, and 21-d SSF was isolated and a comparative analysis, using 2D-PAGE, was performed. The spot pattern proved to be highly reproducible in replicate cultures. Gels from control cultures (without fungus) did not show any spot (data not shown). Most proteins focused in a pH range of 3–6 and had molecular masses from 37 to 100 kDa, a profile similar to those reported for other basidiomycetes [14]. The evolution of enzymes release, concerning both the number of different molecular species and the amount of the proteins detected at different growth stages, can be observed by simple visual inspection of the gels images (Figure 1a-c). The one from the 21-d secretome did not only display the maximum spots number, but also contained all spots detected in the gels of samples from 7-d and 14-d SSF. Then, the spots from the 21-d gel (Figure 1c), were chosen to be excised, in-gel digested, and subjected to MS/MS analysis for protein identification.

**Table 1:** Summary of wheat straw components degradation, digestibility, and sugar yields after biopretreatment processes

		**Loss (% initial content)**			**Digestibility (%)**		**Sugar yields (%)**	
	**Days**	**CEL**	**HEM**	**LIG**	**CEL**	**HEM**	**GLU**	**XYL**
***I. lacteus***	7	9±0	13±7	11±0	28±4	32±8	24±2	28±7
***I. lacteus***	14	17±1	13±4	27±1	56±6	71±5	46±1	61±1
***I. lacteus***	21	21±2	23±2	36±1	78±4	78±2	62±2	61±4
***I. lacteus*+ Mn**^**2+**^	21	18±1	45±6	38±1	82±3	99±8	68±2	55±6
***P. chrysosporium***	21	35±0	70±24	0±0	15±0	22±0	9±0	7±6
***P. ostreatus***	21	22±1	52±13	27±1	55±3	52±8	42±3	30±9
Control*	21	0±0	0±0	0±0	36±6	35±5	36±6	35±5

Cellulose (CEL), hemicellulose (HEM), and lignin (LIG) losses from wheat straw pretreatment (SSF cultures) with *I. lacteus* (different times and Mn^2+^ supplementation), *P. chrysosporium* and *P. ostreatus* are depicted*.* Cellulose and hemicellulose digestibilities (from sugar release referred to the biotreated material) and glucose (GLU) and xylose (XYL) recoveries (as fermentable sugars referred to the initial straw) are also shown. From Salvachúa *et al*. [3,7].

^*^Non-biopretreated wheat straw.

Most of the hits that allowed the identification of *I. lacteus* proteins were from Uniprot (Table 2). Concerning the secretome composition over the time, 70% of the spots were already produced during the first week of incubation (Figure 1a). The major enzymes at this stage (Table 2) were involved in cellulose (endoglucanases and exocellulases), hemicellulose (acetyl xylan esterases and endo-1,4-β-xylanase), and protein degradation. It is worth pointing out that for complete cellulose degradation, the concerted action of three cellulolytic activities is required: endoglucanases, which hydrolyze internal 1,4-β-bonds; cellobiohydrolases I and II (CBHI and CBHII), which act in a synergistic way as exo-cellulases from the reducing and the non-reducing end of the glucidic chain, respectively, releasing cellobiose [15]; and β-glucosidases, which finally break cellobiose into two glucose monomers [13]. The latter enzyme was not detected in these 2D-gels. The acetyl xylan esterase cleaves acetyl side groups from the hetero-xylan backbone and endo-1,4-β-xylanase hydrolyzes internal 1,4-β bonds of xylan [22]. As occurred with cellulose, these enzymes cannot degrade hemicellulose or xylan polymers completely since β-xylosidases, arabinofuranosidases, or α-glucuronidases, which are also required [23], were not produced by the fungus. Finally, proteases, as polyporopepsin, have been implicated in the activation of cellulases, in the cleavage of functional domains of cellobiose dehydrogenases (CDH) [24], and also in trapping nitrogen in lignocellulose under nitrogen-starvation conditions [14].

**Table 2:** **Protein identification from 2D-gel spots from *I. lacteus *secretomes in SSF and SmF cultures**

**Spot**	**Day**^**1**^	**SmF**^**2**^	**Mn**^**3**^	**Predicted protein function**	**Species**^**4**^	**Protein ID**	**MM (kDa)**	**pI**	**Score**	**UP**^**5**^
**1**	7	+	+	Cellobiohydrolase II	IL	B2ZZ24	47.2	5.3	20.6	4
**2**	14	+	+	Cellulase	IL	Q9Y724	55.8	4.6	13.9	3
**3**	7		+	Rhamnogalacturonan-hydrolase	IL	B6E8Y7	46.7	6.9	17.4	3
**4**	7	+	+	Cellobiohydrolase	IL	Q75NB5	54.8	5.3	15.4	4
**5**	14	+	+	Cellulase	IL	Q9Y724	55.8	4.6	215.8	10
**6**	14	+	+	Cellulase	IL	Q9Y724	55.8	4.6	85.3	7
**7**	7			Cellulase	IL	Q9Y724	55.8	4.6	139.4	11
**8**	7	+	+	Cellobiohydrolase	IL	Q75NB5	54.8	5.3	66.7	8
**9**	14			Cellobiohydrolase II	IL	B2ZZ24	47.2	5.3	90.9	7
**10**	7	+	+	Cellobiohydrolase II	IL	B2ZZ24	47.2	5.3	46.0	7
**11**	7	+	+	Cellobiohydrolase II	IL	B2ZZ24	47.2	5.3	25.2	5
**12**	7	+	+	Endoglucanase	IL	Q5W7K4	42.2	4.9	15.4	3
**13**	7	+	+	Acetyl xylan esterase	PC	H2ESB9	38.9	6.5	12.9	2
**14**	7	+	+	Acetyl xylan esterase	PC	H2ESB9	38.9	6.5	15.5	2
**15**	7	+	+	Polyporopepsin	IL	P17576	35.0	4.7	20.0	5
**16**	7	+	+	Aspartic protease	PN	G3XKT3	42.8	5.5	70.0	2
**17**	14		+	Rhamnogalacturonan-hydrolase	IL	B6E8Y7	46.7	6.9	27.3	6
**18**	7	+	+	Acetyl xylan esterase	PC	H2ESB9	38.9	6.5	27.9	2
**19**	7		+	Cellobiohydrolase II	IL	B2ZZ24	47.2	5.3	4.1	2
**20**	7		+	Endo-1,4-β-xylanase A	PC	Q9HEZ1	43.5	5.4	3.1	1
**21**	14		+	Putative protein hypP2	MP	Q6U7U4	47.9	8.7	6.1	1
**22**	14		+	Putative protein hypP2	MP	Q6U7U4	47.9	8.7	6.0	1
**23**	14	+	+	Aspartic protease	PN	G3XKT3	42.8	5.5	3.9	1
**24**	14	+		Aspartic protease	PN	G3XKT3	42.8	5.5	3.7	1
**25**	14	+		Putative protein	PG	E3JYE0	21.2	6.3	2.7	1
**26**	7		+	Aspartic protease	PN	G3XKT3	42.8	5.5	3.9	1
**27**	7		+	Serine-type peptidase^**6**^	PS	Punst1 106327	59.2	4.9	53.1	1
**28**	7		+	Putative protein hypP2	MP	Q6U7U4	47.9	8.7	5.6	1
**29**	7	+	+	Cellulase	IL	Q9Y724	55.8	4.6	9.1	2
**30**	7	+	+	Cellobiohydrolase	IL	Q75NB5	54.8	5.3	2.7	1
**31**	7	+		Cellobiohydrolase	IL	Q75NB5	54.8	5.3	12.9	2
**32**	7	+		Cellobiohydrolase	IL	Q75NB5	54.8	5.3	6.3	2
**33**	7	+	+	Cellobiohydrolase II	IL	B2ZZ24	47.2	5.3	14.2	3
**34**	7		+	Cellobiohydrolase II	IL	B2ZZ24	47.2	5.3	8.5	1
**35**	7		+	Histone H4 (Fragment)	MP	E2LLY3	8.8	11.6	5.1	1
**36**	21			Cellobiohydrolase II	IL	B2ZZ24	47.2	5.3	37.1	6
**37**	21	+		Endoglucanase	IL	Q5W7K4	42.2	4.9	63.1	3
**38**	L	+		GH3/ β-glucosidase	SP	F8PMW3	78.3	4.7	63.3	4
**39**	L	+		Putative protein	MP	E2M3P0	13.2	4.7	3.4	1
**40**	L	+		Glyoxal oxidase^6^	PS	Punst1 68820	59.6	5.2	1.9	1
**41**	L	+		Polyporopepsin	IL	P17576	35.0	4.7	25.6	2
**42**	L	+		Exo-β-(1→3)-galactanase	IL	B9ZZS1	16.8	47.8	6.7	4
**43**	L	+		Mannose-6-phosphatase	PC	Q281W3	38.4	6.6	71.6	4

Only the hits with maximal score from Uniprot database are shown (together with two from JGI).

(1) SSF incubation time in which the spot was first detected and differential spots for SmF cultures (L). (2) and (3) + indicates presence of the spot in SmF, and SSF cultures supplemented with a manganese salt, respectively. (4) Species where the protein had been found: IL *Irpex lacteus,* MP *Moniliophthora perniciosa*, PC *Phanerochaete chrysosporium,* PN *Pholiota nameko*, PO *Pleurotus ostreatus*, PS *Punctularia strigosozonata*, and SL *Serpula lacrymans*. (5) Unique proteins. (6) Proteins identified only using the JGI database.

After the second SSF week, almost all spots from the major proteins detected at the final incubation period were visualized (Figure 1b). Rhamnogalacturonan-hydrolase (spot 17) and different isoforms of cellulases (spots 2, 5, 6, 9) were produced. A very faint spot from pectinase was first observed in 7-d gels (spot 3), but an intense secretion of this protein was detected from two incubation weeks onwards in spite of the fact that pectin content in lignocellulosic materials is low. In parallel, many small-sized proteins came into view. The JGI database matched some of them (spots 21, 22, 25, and 28) to cerato-platanin or Barwin-related endoglucanases. These proteins, secreted by a number of non-pathogenic and pathogenic fungal species when interacting with plant or animal cells, are involved in cell wall biogenesis or degradation [25].

In the 21-d secretome, two new spots (36, 37) were observed, matching respectively with CBHII and endoglucanase (Table 2). A total of 37 spots and 18 different proteins were identified. The protein spot intensity of some cellulases (spots 2, 5, 6, 7, 8, 9, and 11) and an aspartic proteinase (spot 16), probably similar to the polyporopepsin and other aspartic proteases already detected in the 14-d SSF samples, were much more intense in the 21-d sample.

The proteins identified using both databases (Additional file 1: Table S1) are in good agreement in most cases, and the presence of several proteins in some spots insufficiently separated in the gel can be inferred from the data displayed in this table. For example, spot 10, heavily stained in all gels (Figure 1a-c) probably contains a mixture of proteins. The hits returned with maximal scores from JGI and Uniprot corresponded to manganese peroxidase (MnP) and CBHII, respectively, suggesting that both proteins are present in the crude and migrate together. MnP causes the cleavage of C-C and C-O bonds between phenolic lignin units, and this enzyme activity is frequently found in lignocellulose-degradation processes by *I. lacteus*[26].

#### *Analysis of the 21-d* I. lacteus *EPP*

**Figure 2:**
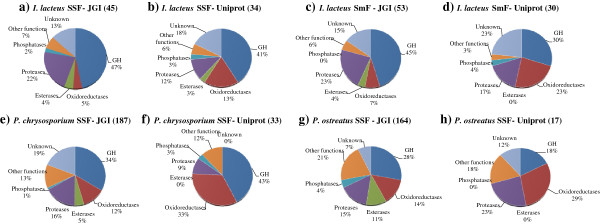
**Functional classification of the lignocellulose-degrading enzymes found in the secretomes, according to Uniprot and JGI searches.** Groups of proteins released by I. lacteus grown on wheat straw **(a-b)** or in submerged cultures **(c-d)**, and in SSF cultures P. chrysosporium **(e-f)** and P. ostreatus **(g-h)**. The total number of protein matches from JGI and Uniprot databases (Additional file 1: Tables S2-S9) is shown in parenthesis. Basidiomycota databases were used for *I. lacteus* secretome searches. The results from *P. chrysosporium* and *P. ostreatus* were searched against their own databases. SSF= solid state fermentation on wheat straw; SmF= submerged cultures in CSS; GH= glycosil hydrolases.

The analysis of the secretome released after growing *I. lacteus* on wheat straw for 21-d using a shotgun proteomics approach was an excellent complement to confirm the data from 2D-gels and disclose the presence of extracellular proteins virtually undetectable by other techniques. The results from the search against the basidiomycota database of Uniprot (Additional file 1: Table S2) identified 34 different proteins, of which 11 hits corresponded to *I. lacteus* enzymes. Most of them are involved in lignocellulose degradation and were functionally classified, according to their biological role, such as glycoside hydrolases (GHs), oxidoreductases, esterases, proteases, phosphatases, and proteins with other or unknown functions (Figure 2). The 45 hits identified from the search using the JGI database (Additional file 1: Table S3) corresponded to enzymes from related basidiomycetes, with similar functionalities to those returned by Uniprot.

Table 3 summarizes the ten extracellular proteins (Top-10) identified with maximal scores from Uniprot database. This set of proteins rather agrees with the most intense spots in 2D-gels (e.g. CBHII, cellulases, and proteases had the highest scores and spot intensities, respectively) and was in accordance with previous reports comparing both methodologies [27]. However, some new proteins were identified that may be relevant for enzymatic decay of wheat straw. Among them, a Mn^2+^-oxidizing and melanin-decolorizing enzyme (MnP) [28], an exo-β-1,3-galactanase, implicated in hemicellulose degradation and isolated by Tsumuraya et al. [29] from this fungus, and a hypothetical peroxidase (cpop21) from a member of the Polyporaceae family, can be highlighted. A recent report from Salvachúa et al. [30] described the purification from *I. lacteus* liquid cultures of a dye-decolorizing peroxidase (DyP), able to degrade azo- and antraquinone-dyes and phenolic and non-phenolic compounds. The protein was analyzed by MALDI-TOF-MS/MS, giving 95% homology with cpop21. Our results here show for the first time DyP production during SSF on wheat straw, a natural lignocellulosic environment.

**Table 3:** Summary of the ten extracellular proteins (Top-10) identified with maximal scores from the shotgun analysis

**EPP**	**Predicted protein function**	**Species**^**1**^	**ID**	**MM (kDa)**	**pI**	**Score**	**UP**^**2**^
***I. lacteus***	Cellobiohydrolase II	IL	B2ZZ24	47.2	5.3	606.0	13
**SSF**	Polyporopepsin	IL	P17576	35.0	4.7	307.2	8
	Cellulase	IL	Q9Y724	55.8	4.6	193.3	9
	Acetyl xylan esterase	PC	H2ESB9	38.9	6.5	142.2	2
	Melanin-decolorizing enzyme (MnP)^3^	Ce	B3IWB3	38.3	5.1	142.1	10
	Peroxidase cpop21 (DyP1)^4^	IL	P87212	53.9	5.0	122.2	8
	Endoglucanase	IL	Q5W7K4	42.2	4.9	118.3	3
	Cellobiohydrolase	IL	Q75NB5	54.8	5.3	106.0	11
	Exo-β-(1→3)-galactanase	IL	B9ZZS1	47.8	6.7	99.8	8
	Rhamnogalacturonan-hydrolase	IL	B6E8Y7	46.7	6.9	84.5	10
***I. lacteus***	Polyporopepsin	IL	P17576	35.0	4.7	4867.5	13
**SmF**	Peroxidase cpop21 (DyP1)^4^	IL	P87212	53.9	5.0	684.8	13
	Melanin-decolorizing enzyme (MnP)^3^	Ce	B3IWB3	38.3	5.1	368.6	11
	Exo-β-(1→3)-galactanase	IL	B9ZZS1	47.8	6.7	202.2	11
	Ribonuclease T2	IL	Q8LW55	41.8	5.1	137.4	4
	Glycoside hydrolase family 3	SL	F8NLG7	89.6	5.0	130.3	1
	Glycoside hydrolase family 3	SL	F8PMW3	78.3	4.7	111.6	2
	Mannose-6-phosphatase	PC	Q281W3	38.4	6.6	87.5	3
	Aspartic protease	PN	G3XKT3	42.8	5.5	63.7	3
	Manganese peroxidase	LG	H2D7E4	38.6	4.5	56.7	3
***P. chrysosporium***	Manganese peroxidase 3	PC	Q1K9D0	39.8	4.6	348.7	8
**SSF**	Glucan 1,3-β-glucosidase	PC	Q2Z1W1	82.0	5.8	327.6	8
	Cellulase	PC	Q7LIJ0	53.8	4.9	286.6	13
	Copper radical oxidase	PC	Q0ZKA8	67.8	5.5	265.8	11
	Endo-1,4-β-xylanase A	PC	Q9HEZ1	43.5	5.4	209.8	4
	Cellobiohydrolase II (fragment)	PC	H3K419	46.3	5.1	189.6	6
	Endo-1,4-β-xylanase C	PC	B7SIW2	42.3	4.9	151.9	6
	Family S53 protease	PC	Q281W2	58.4	4.9	133.8	3
	Exoglucanase 1	PC	P13860	54.8	5.5	117.1	7
	Endo-β-glucanase	PC	C6H0M6	33.6	5.4	113.8	6
***P. ostreatus***	Versatile peroxidase 2 (VP2)^5^	PO	G8FPZ2	38.5	4.7	629.7	8
**SSF**	Subtilisin-like protease	PO	Q6ZYK6	93.2	5.3	330.3	11
	Laccase	PO	Q96TR4	57.4	6.1	284.1	9
	Putative protein	PO	D2JY75	27.8	6.6	162.8	7
	Peptidyl-Lys metalloendopeptidase	PO	P81055	17.9	6.2	118.5	5
	Ribonuclease T2	PO	Q75NB1	41.5	6.4	65.1	4
	α-L-arabinofuranosidase	PO	G0TES6	68.9	8.1	52.2	3
	Putative aspartyl-proteinase (fragment)	PO	Q96TV7	18.5	6.2	51.8	3
	Cellulose 1,4-β-cellobiosidase	PO	A5AA53	49.3	5.6	50.6	4
	Peptidase 1	PO	C4PFY6	38.7	8.2	42.4	2

Uniprot database was used for protein identification from the secretomes of the different fungi in SSF and SmF.

(1) Species where the protein had been found. Abbreviations are those used in Table 2, and: Ce *Ceriporiopsis* sp. and LG *Lenzites gibbosa.* (2) Unique proteins. (3) Nagasaki *et al*. [28]. (4) Salvachúa *et al*. [30]. (5) Camarero *et al*. [31].

Several additional proteins were also detected in the EPP of *I. lacteus*. The most significant were glyoxal oxidase (which is a copper radical oxidase) and CDH (Additional file 1: Tables S2 and S3). These two enzymes are oxidoreductases able to produce the H_2_O_2_ required for the action of extracellular peroxidases [17]. Moreover, CDH has also been implicated in: i) generating highly-reactive hydroxyl radicals *via* Fenton chemistry [15]; ii) binding cellulose, which probably enhances cellulase activities by relieving product inhibition [24]; and iii) preventing phenoxy radical-dependent re-polymerization of lignin [13]. Finally, other hits corresponded to putative uncharacterized proteins whose functions, still unknown, could be assumed to be related to lignocellulose degradation.

### Secretome of *I. lacteus* growing on different culture conditions

#### *Mn*^*2+*^*-supplemented wheat straw*

The supplementation with Mn^2+^ during *I. lacteus* pretreatment is known to improve the enzymatic hydrolysis yields of wheat straw (Table 1). In this study, a 2D-PAGE differential analysis of the enzymes secreted by *I. lacteus,* growing for 21-d on wheat straw supplemented or not with a Mn^2+^ salt, was performed (Figure 1d and 1c). The enzymatic profiles were similar in both cases, and new spots were not observed. However, some proteins were missing in cultures with added Mn^2+^. Most of them (spots 7, 9, 31, 32, 36, 37) corresponded to cellulose-degrading enzymes. In parallel, spot 15, which was identified as polyporopepsin in 2D-gels of 21-d SSF basal cultures, was intensely stained indicating either an increased secretion of this protein or the release of a different enzyme that co-migrated with the peptidase. This spot was analyzed and a mixture of polyporopepsin (similar to that of *Pholiota nameko*) and MnP (similar to that of *Lenzites gibbosa*) was identified with scores around 50, indicating that the latter enzyme was induced by Mn^2+^. In addition, the MM and pI of the overexpressed MnP protein coincided with those reported for a MnP from *I. lacteus*[26]. Surprisingly, the increase of MnP production did not enhance significantly lignin degradation. On the contrary, an extensive hemicellulose loss was observed in these cultures, which could be due to the unspecific degradation of this polymer through Mn^2+^-mediated oxidative reactions [32].

#### *Submerged cultures*

The enzyme production in a non-lignocellulosic medium (CSS) under SmF culture conditions was studied at long incubation times (21 d) mimicking N- and C-limitation, using the same proteomic tools applied for SSF secretomes. The enzyme pattern in SmF (Figure 1e) and SSF (Figure 1c), revealed the differences between the two culture conditions. Whereas many cellulases, CBHs, and proteases were produced under both conditions, six new intense spots emerged in SmF (spots 38–43), whose identities are given in Table 2. Among them, the most heavily stained (spot 38) matched with a β-glucosidase. In contrast, the spots corresponding to rhamnogalacturonan-hydrolases, endo-1,4-β-glucanases, endo-1,4-β-xylanases, some cellulases, and CBHII were missing in SmF cultures (Table 2). Obviously, the production of such a wide battery of cellulases and hemicellulases is not required in a medium without lignocellulose. As expected, spots 21, 22, and 28 from ceratoplatanins neither appeared.

NanoLC-MS/MS identification of the proteins from the whole EPP is presented in Additional file 1: Tables S4 and S5 and the ten extracellular proteins identified with maximal scores (Top-10) using Uniprot database are gathered in Table 3. Some of these enzymes are specially interesting: (1) polyporopepsin, which can be implicated in protein degradation, supplying nitrogen for fungal growth, (2) mannose-6-phosphatase, which has been shown to be involved in the extracellular dephosphorylation of enzymes in carbon-starved cultures [33] and ribonuclease T2, that can be excreted in response to phosphate starvation for phosphate scavenging from RNA [34], and (3) the peroxidase cpop21 (currently identified as DyP), which can be produced to oxidize more complex molecules to be used as energy/nutrient source. Interestingly, and although not included in the Top-10, a second DyP isoenzyme related to that of *Marasmius scorodonius*[35] was detected. Some of these enzymes have probably been secreted as a fungal response to survive in cultures (21 d) limited in essential nutrients. A similar behavior has been previously reported for *Pleurotus sapidus* in SmF cultures, being peptidolytic and ligninolytic enzymes among the major components of its secretome [17]. Two Mn-oxidizing peroxidases, the so-called melanin-oxidizing enzyme mentioned above and one MnP, were also induced in the SmF cultures.

Although the relative representation of each enzyme family (%) in SSF and SmF cultures (Figure 2) was very similar (Additional file 1: Tables S2-S4), the individual proteins were quite different. Comparable expression patterns were described [14] for *P. chrysosporium* growing in SmF (containing cellulose) and SSF cultures on wood. In contrast, Zorn et al. [17] reported that the production of ligninolytic enzymes by *P. sapidus* was influenced by the presence of lignocellulosic inductors.

### Comparative study of the *I. lacteus, P. ostreatus*, and *P. chrysosporium* secretomes growing on wheat straw

#### Fungi, degradation patterns, and databases

The secretome from 21-d *I. lacteus* SSF cultures was compared to those from two white-rot fungi, *P. chrysosporium* and *P. ostreatus*. The growth of these species on wheat straw produced different degradation patterns, and the biotreated material gave sugar yields lower than those attained for *I. lacteus* after enzymatic hydrolysis (Table 1). The MS/MS data from their whole EPP were searched against JGI and Uniprot (Additional file 1: Tables S6-S9). In all cases, the JGI database returned more hits than Uniprot (Figure 2). This is because many hypothetical proteins, deduced from genomic sequences already available, are deposited in that database. Moreover, the percentages yielded for some protein groups were quite different when the inputs from both databases were compared. This is probably due to the fact that many proteins from the JGI have not yet been annotated and may need to be corrected. Even higher differences were found when the number of proteins identified was compared to those predicted from genomes. A total of 769 proteins have been predicted to be part of *P. chrysosporium* secretome [33]. However, in the current work, 4-fold fewer proteins were detected (around 191). This finding highlights the need of studying secretomes from cultures and not by computational predictions, since the protein set released to the extracellular medium is variable and strongly depends on the environment.

The percentages for the diverse functional groups returned by Uniprot (Figure 2) moderately correlated with the different fungal degradation patterns. *P. chrysosporium*, which showed a preferential consumption of carbohydrates during biopretreatment of wheat straw (Table 1), produced a wide battery of GHs comprising all the enzymatic set involved in the complete degradation of cellulose and xylan, which suggests that it could be used for improving enzymatic saccharification of wheat straw. This enzymatic profile seemed to be more similar to those from some plant pathogens (e.g. *Fusarium verticilloides* and *Ustilago maydis*) than to those from the saprophytes included in this study [10,11]. On the contrary, *P. ostreatus* produced less GHs than oxidoreductases (Figure 2H), what can be related to the selectivity towards lignin degradation of *Pleurotus* species [3].

Finally, *I. lacteus,* inducing the simultaneous degradation of all lignocellulosic components (Table 1), released a percentage of GHs similar to *P. chrysosporium,* but the amount of extracellular oxidoreductases was lower. Nevertheless, some proteins were detected in the secretomes from the three fungi such as esterases, proteases, and phosphatases. Proteases, whose role has usually been neglected when studying the decay of lignocellulosic substrates, have been found among the Top-10 proteins of the three fungi, seeming to have a great significance for wheat straw deconstruction.

In view of all these results, it can be concluded that the knowledge on the relative proportions of the different enzyme groups is insufficient to discern the mechanisms implicated in fungal degradation. Detailed information on the identity of the main enzymes belonging to each group is required to elucidate these mechanisms.

#### Oxidoreductases and lignin degradation in wheat straw

As mentioned above, *I. lacteus* secreted DyPs, MnPs, CDHs, and glyoxal oxidases in SSF and SmF cultures (Additional file 1: Tables S2, S3, S4, S5). *P. chrysosporium* released the same enzymes, excluding DyP, but also some others such as lignin peroxidases (LiPs), pyranose 2-oxidase, and GMC oxidoreductases (both producing H_2_O_2_) (Additional file 1: Tables S6-S7). MnPs from *P. chrysosporium* were identified with high scores, although this activity was not previously detected in the soluble fraction from cultures on wheat straw [3], which is in accordance with the scarce lignin degradation produced by this fungus. In contrast, *P. ostreatus* secretome contained mostly versatile peroxidase (VP), MnP, laccases, and glyoxal oxidases (Additional file 1: Table S8-S9). The detection of MnP and laccase activities in the extracellular medium of this fungus, growing on wheat straw, had been previously described [3]. Interestingly, the Top-10 peroxidase in the *P. ostreatus* secretome is VP2, the same isoenzyme found in SSF cultures of the related *Pleurotus eryngii*[31]. Cytochrome P450 and some mono-oxygenases were also identified. These enzymes, whose production has been reported in *Phanerochaete carnosa* growing on spruce and cellulose, participate in the bioconversion of exogenous aromatic compounds [18].

These results suggest that the diversity of lignin-degrading enzymes available in the extracellular matrix does not always run in parallel with the extent of lignin degradation and/or an improvement of the accessibility to carbohydrates in lignocellulose (Table 1). Considering the results obtained for *I. lacteus*, the combination of MnP and DyP activities together with glyoxal oxidases and CDHs, which produce H_2_O_2_ for those peroxidases, seems to be a very effective cocktail for biopretreatment of wheat straw.

#### GHs produced by fungi for wheat straw degradation

Among the carbohydrate active enzymes (CAZY), the GHs (EC 3.2.1.) are the most widespread group and their classification is currently based on sequence similarities. GHs hydrolyze the glycosidic bonds between two or more carbohydrates or between a carbohydrate and a non-carbohydrate moiety. Their accurate identification is sometimes difficult since many families of GHs do not have functional annotations and contain multiple enzymes. The secretomes from *I. lacteus*, *P. chrysosporium* and *P. ostreatus* growing on wheat straw contained enzymes classified into 11, 24 and 30 different GH families, respectively (Table 4). The GHs secreted by *I. lacteus* in SmF cultures cluster into 10 different families. Proteins from some groups, such as GH3, GH5, and GH35 were represented in all of the conditions tested in the present study, regardless of the fungal species or the type of culture. These include a variety of enzymes involved in cellulose and hemicellulose degradation. In contrast, hydrolases from families GH6, GH7, GH10, and GH74 were detected exclusively in SSF cultures. Only these four GH families contain CBHs, suggesting that this type of exocellulases is really induced by lignocellulose.

**Table 4:** Diversity of GH families returned by JGI and Uniprot databases from the secretomes analyzed

**Organism**	**Culture**																**Glycoside hydrolase families**																								
*I. lacteus*	SSF		2	3*		5	6	7		10														35		43*				61			74					92*			
*I. lacteus*	SmF		2	3		5								15								30	31	35		43*												92*			125
*P. chrysosporium*	SSF			3		5	6	7	8	10	11	12		15		17	18	20	25	27	28	30	31	35	37	43	47			61	71						88				
*P. ostreatus*	SSF	1	2	3	4	5	6	7	8	10		12	13	15	16			20		27	28		31	35	37		47	51	55			72		76	78	79	88	92	105	115	
**Type of enzymes**^**1**^** Cellulose degrading enzymes**																																									
Cellulases									X																																
Endo-1,4-β-glucanases				X		X		X				X			X													X					X								
Cellobiohydrolases							X	X		X																							X								
Exo-1,4-β-glucanases		X		X			X																																		
β-Glucosidases		X		X																		X																			
**Hemicellulose degrading enzymes**																																									
Xylanases/xyloglucanases						X				X	X	X				X										X							X	X							
Endo-1,4-β-xylanases				X		X			X						X																										
Endo-1,3-β-xylanases										X																X															
β-Xylosidases																						X				X															
Arabinofuranosidases				X																						X		X													
α- Glucuronidases					X																															X				X	
β-Galactosidases		X	X																					X																	
β-Mannosidases		X	X			X																																			
**Starch degrading enzymes**													X	X									X				X														
**Other GHs **^**2**^		X	X	X	X	X	X	X	X			X				X	X	X	X	X	X	X		X	X	X		X	X	X	X	X		X	X	X	X	X	X	X	X

Crosses (X) show the GH family to which the proteins identified in the different secretomes belong, according to http://www.cazy.org/Glycoside-Hydrolases.html.

(*) These GH families were not detected in *I. lacteus*’ spots, but were identified from EPP analyses.

(^1^) Each functional group contains the most representative enzymes.

(^2^) This group contains activities different to those detailed in the Table.

Many other families of GHs containing starch-degrading enzymes (GH13, GH15, GH31) and pectinases (GH2, GH28) were found in *P. ostreatus*, and several families were only represented in this species. Some of them are GH16, GH55, GH72, GH76, GH78, and GH105, which are mostly implied in fungal metabolism, as for example α-mannosidases [36], and GH4, GH51, GH79, and GH115, which include enzymes such as α-arabinofuranosidases and α-glucuronidases implicated in the complete hydrolysis of hemicellulose [23]. Similarly, proteins from some GH families were represented in *P. chrysosporium* (11, 17, 18, 25, 30, 71), but did not appear in the two other species. Enzymes from the family GH30 are involved in the complete hydrolysis of cellulose and xylan by β-glucosidases and β-xylosidases, what implies the extensive sugar consumption during biopretreatment (Table 1).

The GH families detected only in *I. lacteus* were GH74 in SSF and GH125 in SmF cultures. Proteins from family GH74 have been reported to enhance the performance of complex cell-wall digesting cocktails [37]. The only protein in family GH125 is an exo-α-1,6-mannosidase, an enzyme barely described to date [38]. The number of GH families represented in SmF and SSF *I. lacteus* cultures was similar, although they were quite different from a qualitative point of view. It is worth to emphasize that proteins from family GH30 were detected only in SmF cultures.

The detection of the called “enigmatic” family GH61 in *P. chrysosporium* and *I. lacteus* should also be pointed out, since proteins from this group have been implicated in the initial steps of lignocelluloses breakdown by white-rot fungi, disrupting the cellulose structure and enhancing its digestibility by cellulases in lignocelluloses [39].

### Why *I. lacteus* is so efficient pretreating wheat straw for 2G-ethanol production?

Based on the results presented here, we propose that *I. lacteus* degrades cellulose using a large machinery of exocellulases and endoglucanases. Simultaneously, hemicellulose and pectins are mainly being broken down *via* endo-1,4-β-xylanase and acetyl xylan esterase, and rhamnogalacturonan hydrolase, respectively. Due to the specific hydrolytic action of these enzymes, large polysaccharide fragments are mostly released. Our results also suggest that the enzymatic action of lignin-degrader oxidoreductases such as MnP and DyP, and proteases such as polyporopepsin, enhance wheat straw deconstruction by facilitating the action of the carbohydrate-degrading enzymes. This enzyme profile yielded easily hydrolysable products with high sugar content. The key of that sugar enrichment is that the extracellular enzymatic pool is deficient in those proteins that catalyze the complete hydrolysis of cellulose and hemicelluloses to their monomeric units, hampering extensive sugar consumption for fungal growth. As an example, β-glucosidases, β-xylosidases, and α-glucuronidases, or proteins included in their GHs families (such as GH1, GH3, GH4, GH30, GH43, GH51, and GH115) were not detected in the secretome of *I. lacteus* from SSF cultures, or were detected as minor proteins. The positive effect on glucose yields of adding Mn^2+^ to the cultures could be explained from two findings: the release of several isoforms of cellulase showed some degree of inhibition, which probably caused a decrease in cellulose degradation and consumption, and the induction of MnP that presumably produced an enhancement in cellulose accessibility during the enzymatic hydrolysis.

## Conclusions

The current work describes for the first time the composition of the secretome of *I. lacteus* growing on wheat straw. The protein pattern secreted during SSF fungal growth justifies the fitness of this species for biopretreatment processes in 2G-ethanol production and provides insight into these biological processes. The secretome of *I. lacteus* can be of interest to be used for pretreatment of lignocellulosic material or enzymatic hydrolysis improvement through the preparation of optimized enzyme–cocktails. Due to the potential of *I. lacteus* in these processes, this fungus may warrant consideration in future genome projects.

## Methods

### Fungal strains and culture media

The white-rot fungi used in the present study were obtained from different fungal collections. *I. lacteus* Fr. 238 617/93 was provided from the Culture Collection of Basidiomycetes from the Academy of Sciences of the Czech Republic (CCBAS, Prague). *P. chrysosporium* CBS 481.73 and *P. ostreatus* CBS 411.71 were obtained from the Centraalbureau voor Schimmelcultures (CBS, Baarn, The Netherlands). The fungal species were maintained on 2% malt extract agar (MEA) tubes at 4°C. Prior to the experiments, the three fungi were grown at 28°C during 7 days on MEA plates. Four agar plugs of 1-cm^2^ were excised, inoculated into 250 mL Erlenmeyer flasks with 30 mL of growth medium (pH 5.6) and incubated at 28°C, and 180 rpm for 7 days. The corn-step solids growth medium (CSS) used contained (L^-1^): corn steep solids, 26.3 g; glucose, 40 g; FeSO_4_×7H_2_O, 0.4 g; (NH_4_)_2_SO_4_, 9 g; KH_2_PO_4_, 4 g; CaCO_3_, 7 g. Each culture was aseptically homogenized (Omnimixer, Sorvall), and 2.5 mL were added to 250 mL flasks with 30 mL of CSS, incubating for 5 days as described above. These cultures were used as inocula for (1) solid state fermentation cultures (SSF) of the three fungal species and (2) submerged fermentation (SmF) cultures of *I. lacteus.*

### SSF cultures and secretome extraction

Wheat (*Triticum aestivum*) straw, harvested from Galicia fields (Spain) and composed of 36.9% cellulose and 23% hemicellulose (18% xylan, 3.4% arabinan, 1.1% mannan, and 0.5% galactan), was chopped into fragments smaller than 1 cm. 100 mL Erlenmeyer flasks containing two grams of this substrate and distilled water (6 mL) were autoclaved at 121°C for 15 min, inoculated with 5-day-old mycelium from the different fungi (2 mL) and incubated at 28°C as previously described [3]. Non-inoculated samples were kept under the same conditions to be used as controls. The cultures of *P. ostreatus* and *P. chrysosporium* were collected after 21-d incubation. *I. lacteus* cultures were sampled after 7, 14 and 21 days. In parallel, MnSO_4_ (0.3 mM) was added to wheat straw before autoclaving and then the flasks were incubated with *I. lacteus* for 21-d as detailed above. All cultures were performed in duplicate. After SSF, the cultures were extracted with water (15 mL) at 4°C and 180 rpm for 2 h, and filtered under vacuum to separate the solid fraction from the water-soluble components. Liquid samples were dialyzed by centrifugation with 30 volumes of Milli-Q water using 3-kDa cutoff Amicon Ultra centrifugal filter units (Millipore Corporation) and then freeze-dried for further protein isolation.

### SmF cultures of *I. lacteus* and secretome extraction

Submerged cultures were performed in triplicate in 250 mL flasks with 30 mL CSS. 21-d cultures were harvested and filtered to separate the mycelium. Then the culture broth was vacuum-filtered through 0.22 μm membranes (Millipore Corporation), dialyzed against water under continuous stirring at 4°C in a tangential ultra-filtration system (Amicon, Millipore Corporation) using a 3-kDa cutoff membrane and freeze-dried for further protein isolation.

### Preparation of protein extracts from SSF and SmF cultures

Freeze-dried samples from SSF and SmF samples were resuspended in water and precipitated using the methanol/chloroform protocol to remove salts, sugars and other impurities [40]. Pellets were dried and resuspended in different solutions depending on the subsequent analysis method, as described below. Protein concentration was estimated using the RC DC Protein Assay kit from Bio-Rad.

### Secretome analysis

Two different approaches were followed to study the fungal secretomes: (1) Two dimensional-polyacrylamide gel electrophoresis (2D-PAGE), followed by tryptic digestion of each spot and nanoLC-MS/MS analysis of the peptides and (2) shotgun analysis of the EPP, consisting on the tryptic digestion of the unfractionated EPP and nanoLC-MS/MS analysis of the peptides released.

#### 2D-electrophoresis

Samples from SSF and SmF cultures of *I. lacteus* were individually analyzed in 2D-gels. The instruments, products and methods detailed in this section were those recommended by Bio-Rad unless otherwise stated. Protein pellets were resuspended in a sample solution containing 7 M urea, 2 M thiourea, 4% (w/v) CHAPS, and 0.0003% (w/v) bromophenol blue. For isoelectrofocusing (IEF), 140 μL of sample solution containing around 30 μg total protein, 18.2 mM dithiotreitol (DTT), and 0.5% immobilized pH gradient buffer solution were loaded into 7 cm non linear pH 3–10 strips.

The first dimension was run in a Protean IEF Cell system. After IEF, the strips were equilibrated, and the focused proteins reduced and alkylated, by immersion for 15 min in 2 mL equilibration buffer (50 mM Tris–HCl pH 8.8, 2% [w/v] sodium dodecylsulfate [SDS], 6 M urea, 30% [v/v] glycerol) containing 52 mM dithiothreitol (DTT), and then for 15 min the same buffer containing 130 mM iodoacetamide. The strips were applied on 12% SDS-gels and the second dimension was run in a cooled Mini-Protean 3 Dodeca Cell at 0.5 watts/gel for 30 min and then at 1.5 watts/gel until the die-front reached the bottom edge (approximately 1 h). As molecular mass markers, 2 μL Precision Plus Protein Unstained Standards were used.

Gels were stained with SYPRO Ruby protein gel stain. An EXQuest Spot Cutter was used for image acquisition and spots picking. Gel pieces (1 mm^2^) from 2D-spots of the 21-d SSF *I. lacteus* secretome (Figure 1c) and the differential spots from Mn^2+^-supplemented cultures (Figure 1d) and submerged cultures (Figure 1e) were excised. Fragments were rehydrated for 45 min at 4°C with a solution containing 12.5 ng/μL sequencing grade modified trypsin (Promega) in 50 mM ammonium bicarbonate, and then incubated overnight at 30°C in the same solution. The supernatant was removed and kept, and the fragments washed for 20 min at room temperature with 100% acetonitrile and then with 0.5% trifluoroacetic acid (TFA). All supernatants were pooled together, dried by vacuum centrifugation and reconstituted in 0.1% TFA.

#### EPP analysis

The secretomes from *I. lacteus*, *P. ostreatus*, and *P. chrysosporium* 21-d SSF cultures, and from submerged cultures of *I. lacteus*, contained variable amounts of pigmented substances that could interfere with the LC-MS/MS analysis of the EPP. To clean the samples, the protein pellets were dissolved in sample buffer (37.5 mM Tris–HCl pH 8, 1.5% [w/v] SDS, 1 mM EDTA, 1.96 mM DTT, 0.005% [w/v] bromophenol blue and 12.5% [v/v] glycerol). Aliquots containing around 5 μg of protein in a total volume of 15 μL were denatured at 100°C for 15 min and run into a 12% SDS-gel. Prestained molecular mass markers were run in parallel. All markers were individually visualized after a short run of approximately 10 min at 25 mA in the stacking gel and 7 min at 20 mA in the resolving gel, and then the electrophoresis was stopped and the gel stained with Colloidal Blue Stain (Invitrogen). The protein gel fragment was horizontally cut into 3 similar fragments, which were excised into small pieces (1 mm^2^), destained, and reduced and alkylated as previously described. After washing and drying, the three samples were separately digested with trypsin as explained before, and then pooled again to analyze the tryptic peptides mixture. Prior to identification, samples were purified with C18-ZipTips (Millipore Corporation), eluting with 70% acetonitrile in 50 mM ammonium bicarbonate, and dried by vacuum centrifugation.

#### Peptide analysis by nanoLC-MS/MS

Peptide mixtures from enzymatic digestions were dissolved in 5 μL solution A (0.1% formic acid in 2% acetonitrile), and analyzed by nanoLC-MS/MS in a nanoEasy-HPLC (Proxeon) coupled to a nanoelectrospay ion source (Proxeon). Peptides were loaded onto a C18-A1 2 cm-precolumn (Thermo Scientific EASY-Column) and then eluted onto a Biosphere C18 capillary column (inner diameter 75 μm, 16 cm long, 3 μm particle size, Nanoseparations) at a flow-rate of 250 nL/min using solutions A and B (0.1% formic acid in pure acetonitrile). Spots from 2D-gels were separated using the following gradient: 40 min from 0-35% Buffer B, and 5 min from 35-45% Buffer B. For shotgun analysis, the peptides from EPP digestions were eluted with a gradient including 55 min from 0-35% Buffer B, and 16 min from 35-45% Buffer B. Full-scan MS spectra (m/z 300–1700) were acquired on an LTQ-Orbitrap Velos (Thermo Scientific) in the positive ion mode. The 15 most intense ions were selected for collision induced dissociation (CID) fragmentation in the LTQ Velos. Mass spectra files were searched against databases from Uniprot (http://www.uniprot.org/) and the Joint Genome Institute (JGI) (http://genome.jgi.doe.gov/programs/fungi/index.jsf) using the SEQUEST and MASCOT search engines through Proteome Discoverer (version 1.3.0.339, Thermo). Basidiomycota databases from Uniprot and JGI were used for *I. lacteus* homology queries since there is not a complete database of this fungus. In contrast, specific databases of *P. chrysosporium* and *P. ostreatus* from JGI and Uniprot were used for further peptide identification. Search parameters included a maximum of two missed cleavages allowed, carbamidomethylation of cysteines as a fixed modification and oxidation of methionine as a variable modification. The peptides were validated through the algorithm Percolator (FDR 0.05) and only those with high and medium confidence were admitted. Unless otherwise specified, protein identifications were accepted if they contained at least two identified peptides.

## Abbreviations

CBH: Cellobiohydrolase; CDH: Cellobiose dehydrogenase; DyP: Dye-decolorizing peroxidase; EPP: Extracellular pool of proteins; GH: Glycoside hydrolase; IEF: Isoelectric focusing; LiP: Lignin peroxidase; MM: Molecular mass; MnP: Manganese peroxidase; nanoLC-MS/MS: nano liquid chromatography-tandem mass spectrometry; pI: Isoelectric point; SDS: Sodium dodecylsulfate; SmF: Submerged fermentation; SSF: Solid state fermentation; VP: Versatile peroxidase.

## Competing interests

The authors declare that they have no competing interests.

## Authors’ contributions

DS participated in the design of the study, carried out the experiments, organized and interpreted the data, and drafted the manuscript. MT made substantial contributions to conception of the initial experiments. MF, FG-T, and VR performed the proteomics experiments. ATM revised critically the manuscript with substantial contribution to its intellectual content. MJM and AP participated in the design, coordination, and data analysis of the study, and corrected the manuscript. All authors read and approved the final manuscript.

## Supplementary Material

Additional file 1: Table S1 Protein identification from 2D-gels of the *I. lacteus* secretomes under SSF and SmF conditions. All matches returned by JGI and Uniprot are given, ordered according to maximal score in each database. UP= Unique peptides; *Proteins already annotated in JGI. **Tables S2 and S3**. Functional classification of proteins from the complete secretome of *I. lacteus* growing on wheat straw after search against the Basidiomycota databases of Uniprot (S2) and JGI (S3). **Tables S4 and S5**. Functional classification of proteins from the complete secretome of *I. lacteus* growing on CSS SmF cultures after search against the Basidiomycota databases of Uniprot (S4) and JGI (S5). **Tables S6 and S7**. Functional classification of proteins from the secretome of *P. chrysosporium* growing on wheat straw, after search against the Basidiomycota databases of Uniprot (S6) and JGI (S7). **Tables S8 and S9**. Functional classification of proteins from the secretome of *P. ostreatus* growing on wheat straw after search against the Basidiomycota databases of Uniprot (S8) and JGI (S9). Click here for file
