# Supplementary material for: Differential proteomic analysis of the secretome of Irpex lacteus and other white-rot fungi during wheat straw pretreatment
Source: Biotechnol Biofuels. 2013 Aug 10;6:115. doi: 10.1186/1754-6834-6-115 (PMC3750859; doi:10.1186/1754-6834-6-115)
Supplement: Additional file 1: Table S1 — Protein identification from 2D-gels of the I. lacteus secretomes under SSF and SmF conditions. All matches returned by JGI and Uniprot are given, ordered according to maximal score in each database. UP= Unique peptides; *Proteins already annotated in JGI. Tables S2 and S3. Functional classification of proteins from the complete secretome of I. lacteus growing on wheat straw after search against the Basidiomycota databases of Uniprot (S2) and JGI (S3). Tables S4 and S5. Functional classification of proteins from the complete secretome of I. lacteus growing on CSS SmF cultures after search against the Basidiomycota databases of Uniprot (S4) and JGI (S5). Tables S6 and S7. Functional classification of proteins from the secretome of P. chrysosporium growing on wheat straw, after search against the Basidiomycota databases of Uniprot (S6) and JGI (S7). Tables S8 and S9. Functional classification of proteins from the secretome of P. ostreatus growing on wheat straw after search against the Basidiomycota databases of Uniprot (S8) and JGI (S9). [file 1754-6834-6-115-S1.pdf]

Additional file 1

Table S1. Protein identification from 2D-gels of the *I. lacteus* secretomes under SSF and SmF conditions. All matches returned by JGI and Uniprot, ordered according to maximal score in each database, are given. UP= Unique peptides; CBM= carbohydrate-binding module. *Proteins already annotated in JGI.

	**Homologous to proteins**	**Database**	**Species**	**Accession number**	**MM (kDa)**	**pI**	**Score**	**Proteins**	**UP**	**Peptides**	**Coverage (%)**
**Spot 1**	GH10*	JGI	*Bjerkandera adusta*	Bjead1_1 24950	40.2	6.5	**5.3**	4	2	2	5.6
	Aspartic-type endopeptidase	JGI	*Sphaerobolus stellatus*	Sphst1 174318	35.0	4.8	**5.0**	5	2	2	3.3
	Cellobiohydrolase II	Uniprot	*Irpex lacteus*	B2ZZ24	47.2	5.3	**20.6**	1	4	4	12.6
	Endoglucanase	Uniprot	*Irpex lacteus*	Q5W7K4	42.2	4.9	**19.0**	1	3	3	11.0
	Polyporopepsin	Uniprot	*Irpex lacteus*	P17576	35.0	4.7	**14.1**	1	5	5	13.8
	Cellulase	Uniprot	*Irpex lacteus*	Q9Y724	55.8	4.6	**12.1**	1	4	4	9.1
	Cellobiohydrolase	Uniprot	*Irpex lacteus*	Q75NB5	54.8	5.3	**11.8**	2	3	3	8.3
**Spot 2**	Cellulase	Uniprot	*Irpex lacteus*	Q9Y724	55.8	4.6	**13.9**	1	3	3	6.3
	Cellobiohydrolase	Uniprot	*Irpex lacteus*	Q75NB5	54.8	5.3	**8.9**	2	3	3	5.6
	Cellobiohydrolase II	Uniprot	*Irpex lacteus*	B2ZZ24	47.2	5.3	**7.8**	1	3	3	7.3
**Spot 3**	Protein binding. ATP binding (actin)	JGI	*Paxillus involutus*	Paxin1 20222	30.8	5.1	**2.6**	65	1	1	4.0
	Rhamnogalacturonan-hydrolase	Uniprot	*Irpex lacteus*	B6E8Y7	46.7	6.9	**17.4**	1	3	3	9.3
**Spot 4**	β-1.6-N-acetylglucosaminyltransferase	JGI	*Phanerochaete chrysosporium*	Phchr1 121730	89.5	4.6	**30.7**	2	1	1	2.2
	Cellobiohydrolase	Uniprot	*Irpex lacteus*	Q75NB5	54.8	5.3	**15.4**	2	4	4	9.4
**Spot 5**	GH7*	JGI	*Phanerochaete carnosa*	Phaca1 264060	53.8	4.3	**50.1**	6	2	2	4.9
	Cellulase	Uniprot	*Irpex lacteus*	Q9Y724	55.8	4.6	**215.8**	1	10	10	33.1
**Spot 6**	GH7*	JGI	*Phanerochaete carnosa*	Phaca1 264060	53.8	4.3	**17.9**	1	1	1	2.9
	Cellulase	Uniprot	*Irpex lacteus*	Q9Y724	55.8	4.6	**85.3**	1	7	7	23.8
	Peroxidase cpop21 (DyP1)	Uniprot	Polyporaceae	P87212	53.9	5.0	**12.1**	1	1	1	3
**Spot 7**	GH7	JGI	*Sphaerobolus stellatus*	Sphst1 205747	35.8	4.6	**16.7**	48	1	2	8.5
	Cellulase OS	Uniprot	*Irpex lacteus*	Q9Y724	55.8	4.6	**139.4**	1	11	11	34.8
**Spot 8**	GH7	JGI	*Sphaerobolus stellatus*	Sphst1 54354	53.3	5.1	**21.3**	50	2	2	5.6
	GH7*	JGI	*Pleurotus ostreatus*	PleosPC9_1 100231	47.9	4.5	**6.4**	30	2	2	5.3
	Cellobiohydrolase	Uniprot	*Irpex lacteus*	Q75NB5	54.8	5.3	**66.7**	1	5	8	17.9
	Exocellulase	Uniprot	*Irpex lacteus*	Q9Y723	55.0	4.8	**52.0**	1	1	4	10.3
	Cellulase	Uniprot	*Irpex lacteus*	Q9Y724	55.8	4.6	**43.4**	1	6	6	16.2
	Cellulase	Uniprot	*Irpex lacteus*	Q9Y722	54.5	4.9	**41.7**	1	6	6	14.7
**Spot 9**	1. 4-beta cellobiohydrolase. GH6. CBM*	JGI	*Punctularia strigosozonata*	Punst1 89180	47.1	4.9	**36.3**	6	1	1	4.7
	Cellobiohydrolase II	Uniprot	*Irpex lacteus*	B2ZZ24	47.2	5.3	**90.9**	1	7	7	19.9
	Cellobiohydrolase	Uniprot	*Irpex lacteus*	Q75NB5	54.8	5.3	**60.4**	2	6	6	18.0
	Peroxidase cpop21 (DyP1)	Uniprot	Polyporaceae	P87212	53.9	5.0	**3.0**	1	1	1	3.0

Table S1. Continued

**Homologous to proteins**		**Database**	**Species**	**Accession number**	**MM (kDa)**	**pI**	**Score**	**Proteins**	**UP**	**Peptides**	**Coverage (%)**
**Spot 10**	MnP9-short*	JGI	*Phlebia brevispora*	Phlbr1 147108	38.3	4.6	**21.1**	26	2	4	16.4
	MnP2s*	JGI	*Trametes versicolor*	Trave1 112835	38.3	4.5	**19.0**	24	1	3	11.6
	CBM1-GH10*/CBM1	JGI	*Phlebiopsis gigantea*	Phlgi1 85016	43.1	4.9	**13.8**	8	2	2	6.7
	GH7	JGI	*Bjerkandera adusta*	Bjead1_1 207890	22.4	6.3	**9.1**	54	1	2	10.6
	GH7*	JGI	*Pleurotus ostreatus*	PleosPC9_1 100231	47.9	4.5	**6.1**	29	2	2	5.3
	GH7*	JGI	*Phanerochaete carnosa*	Phaca1 264060	53.8	4.3	**5.6**	5	1	2	4.9
	Cellobiohydrolase II	Uniprot	*Irpex lacteus*	B2ZZ24	47.2	5.3	**46.0**	1	7	7	19.9
	Endoglucanase	Uniprot	*Irpex lacteus*	Q5W7K4	42.2	4.9	**32.5**	1	3	3	11.0
	Cellobiohydrolase	Uniprot	*Irpex lacteus*	Q75NB5	54.8	5.3	**29.6**	2	6	6	15.0
	Rhamnogalacturonan-hydrolase	Uniprot	*Irpex lacteus*	B6E8Y7	46.7	6.9	**25.6**	1	7	7	21.7
	Cellulase	Uniprot	*Irpex lacteus*	Q9Y724	55.8	4.6	**20.0**	1	5	5	14.5
	Melanin-decolorizing enzyme (MnP)	Uniprot	*Ceriporiopsis sp.*	B3IWB3	38.3	5.1	**18.3**	13	4	5	22.3
	Manganese peroxidase 1	Uniprot	*Spongipellis sp.*	Q2HWK0	37.8	4.5	**12.1**	1	2	2	9.8
	Manganese peroxidase 3	Uniprot	*Polyporus brumalis*	G0Z9F2	38.1	4.6	**10.9**	20	1	2	9.1
	Endo-1.4-β-xylanase A	Uniprot	*Phanerochaete chrysosporium*	Q9HEZ1	43.5	5.4	**10.1**	2	2	2	6.6
	Cellulase	Uniprot	*Irpex lacteus*	Q9Y722	54.5	4.9	**8.4**	1	2	2	4.6
	Polyporopepsin	Uniprot	*Irpex lacteus*	P17576	35.0	4.7	**5.4**	1	2	2	6.5
	Exo-β-(1→3)-galactanase	Uniprot	*Irpex lacteus*	B9ZZS1	47.8	6.7	**4.4**	1	2	2	7.6
	Peroxidase cpop21(DyP1)	Uniprot	Polyporaceae	P87212	53.9	5.0	**3.0**	1	1	1	3.0
**Spot 11**	CBM1-GH10*	JGI	*Phlebiopsis gigantea*	Phlgi1 85016	43.1	4.9	**9.2**	8	2	2	6.7
	Cellobiohydrolase II	Uniprot	*Irpex lacteus*	B2ZZ24	47.2	5.3	**25.2**	1	5	5	14.8
	Endoglucanase	Uniprot	*Irpex lacteus*	Q5W7K4	42.2	4.9	**17.7**	1	2	2	7.5
	Cellulase	Uniprot	*Irpex lacteus*	Q9Y724	55.8	4.6	**14.6**	1	4	4	10.5
	Melanin-decolorizing enzyme (MnP)	Uniprot	*Ceriporiopsis sp.*	B3IWB3	38.3	5.1	**13.2**	1	4	4	18.4
	Polyporopepsin	Uniprot	*Irpex lacteus*	P17576	35.0	4.7	**11.1**	1	3	3	10.6
	Cellobiohydrolase	Uniprot	*Irpex lacteus*	Q75NB5	54.8	5.3	**10.2**	2	2	2	5.6
**Spot 12**	Peptidase S41	JGI	*Bjerkandera adusta*	Bjead1_1 256345	74.1	5.6	**13.3**	4	1	2	4.3
	Esterase/lipase/thioesterase	JGI	*Phanerochaete chrysosporium*	Phchr1 129015	30.5	6.7	**9.0**	5	2	2	13.2
	Endoglucanase	Uniprot	*Irpex lacteus*	Q5W7K4	42.2	4.9	**15.4**	1	3	3	11.0
	Cellobiohydrolase	Uniprot	*Irpex lacteus*	Q75NB5	54.8	5.3	**11.9**	2	2	2	5.0
	Melanin-decolorizing enzyme (MnP)	Uniprot	*Ceriporiopsis sp.*	B3IWB3	38.3	5.1	**8.7**	1	2	2	9.5
	Cellobiohydrolase II	Uniprot	*Irpex lacteus*	B2ZZ24	47.2	5.3	**8.2**	1	3	3	7.3
	Acetyl xylan esterase	Uniprot	*Phanerochaete chrysosporium*	H2ESB9	38.9	6.5	**9.0**	1	2	2	10.1
**Spot 13**	Esterase/lipase/thioesterase	JGI	*Phanerochaete chrysosporium*	Phchr1 129015	30.5	6.7	**12.9**	5	2	2	13.2
	Acetyl xylan esterase	Uniprot	*Phanerochaete chrysosporium*	H2ESB9	38.9	6.5	**12.9**	1	2	2	10.1
	Endoglucanase	Uniprot	*Irpex lacteus*	Q5W7K4	42.2	4.9	**11.0**	1	3	3	11.0
	Cellobiohydrolase II	Uniprot	*Irpex lacteus*	B2ZZ24	47.2	5.3	**8.3**	1	3	3	7.3
	Melanin-decolorizing enzyme (MnP)	Uniprot	*Ceriporiopsis sp.*	B3IWB3	38.3	5.1	**6.9**	1	2	2	9.5
**Spot 14**	Peptidase S41	JGI	*Bjerkandera adusta*	Bjead1_1 256345	74.1	5.6	**20.2**	5	2	2	4.3
	Esterase/lipase/thioesterase	JGI	*Phanerochaete chrysosporium*	Phchr1 129015	30.5	6.7	**15.5**	5	2	2	13.2
	Acetyl xylan esterase	Uniprot	*Phanerochaete chrysosporium*	H2ESB9	38.9	6.5	**15.5**	1	2	2	10.1
	Endoglucanase	Uniprot	*Irpex lacteus*	Q5W7K4	42.2	4.9	**12.4**	1	2	2	8.8
	Cellobiohydrolase II	Uniprot	*Irpex lacteus*	B2ZZ24	47.2	5.3	**11.2**	1	3	3	7.3
	Melanin-decolorizing enzyme (MnP)	Uniprot	*Ceriporiopsis sp.*	B3IWB3	38.3	5.1	**7.2**	1	2	2	9.5

Table S1. Continued

Homologous to proteins		Database	Species	Accession number	MM (kDa)	pI	Score	Proteins	UP	Peptides (%)	Coverage
**Spot 15**	Aspartic-type endopeptidase activity	JGI	*Sphaerobolus stellatus*	Sphst1 264141	33.9	5.2	**10.9**	7	2	2	6.1
	GH7*	JGI	*Phanerochaete carnosa*	Phaca1 264060	53.8	4.3	**8.7**	12	2	2	4.9
	Polyporopepsin	Uniprot	*Irpex lacteus*	P17576	35.0	4.7	**20.0**	1	5	5	14.1
	Endoglucanase	Uniprot	*Irpex lacteus*	Q5W7K4	42.2	4.9	**17.7**	1	4	4	12.8
	Cellulase	Uniprot	*Irpex lacteus*	Q9Y724	55.8	4.6	**15.8**	1	4	4	9.1
	Cellobiohydrolase II	Uniprot	*Irpex lacteus*	B2ZZ24	47.2	5.3	**13.7**	1	4	4	12.0
	Cellobiohydrolase	Uniprot	*Irpex lacteus*	Q75NB5	54.8	5.3	**10.3**	2	3	3	7.1
**Spot 16**	Aspartic-type endopeptidase activity	JGI	*Sphaerobolus stellatus*	Sphst1 264141	33.9	5.2	**65.8**	3	1	2	6.1
	Aspartic protease	Uniprot	*Pholiota nameko*	G3XKT3	42.8	5.5	**70.0**	1	2	2	6.1
	Endoglucanase	Uniprot	*Irpex lacteus*	Q5W7K4	42.2	4.9	**30.1**	1	3	3	11.0
	Cellobiohydrolase II	Uniprot	*Irpex lacteus*	B2ZZ24	47.2	5.3	**25.5**	1	4	4	13.5
	Polyporopepsin	Uniprot	*Irpex lacteus*	P17576	35.0	4.7	**17.8**	1	4	4	19.7
**Spot 17**	Rhamnogalacturonan-hydrolase	Uniprot	*Irpex lacteus*	B6E8Y7	46.7	6.9	**27.3**	1	6	6	23.7
**Spot 18**	Esterase/lipase/thioesterase	JGI	*Phanerochaete chrysosporium*	Phchr1 129015	30.5	6.7	**19.8**	5	2	2	13.2
	Acetyl xylan esterase	Uniprot	*Phanerochaete chrysosporium*	H2ESB9	38.9	6.5	**27.9**	1	2	2	10.1
	Cellobiohydrolase II	Uniprot	*Irpex lacteus*	B2ZZ24	47.2	5.3	**5.8**	1	2	2	5.1
**Spot 19**	Esterase/lipase/thioesterase	JGI	*Phanerochaete chrysosporium*	Phchr1 129015	30.5	6.7	**5.9**	4	1	1	8.9
	Cellobiohydrolase II	Uniprot	*Irpex lacteus*	B2ZZ24	47.2	5.3	**4.1**	1	2	2	4.7
**Spot 20**	Endo-1.4-β-xylanase A	Uniprot	*Phanerochaete chrysosporium*	Q9HEZ1	43.5	5.4	**3.1**	2	1	1	3.4
**Spot 21**	Cerato-platanin/ Barwin-related endoglucanase	JGI	*Pleurotus ostreatus*	PleosPC9_1 102212	14.8	5.2	**3.3**	1	1	1	10.5
	Putative uncharacterized protein hypP2	Uniprot	*Moniliophthora perniciosa*	Q6U7U4	47.9	8.7	**6.1**	1	1	1	4.7
**Spot 22**	Cerato-platanin/ Barwin-related endoglucanase	JGI	*Pleurotus ostreatus*	PleosPC9_1 102212	14.8	5.2	**10.9**	1	1	1	10.5
	Putative uncharacterized protein	Uniprot	*Melampsora larici-populina*	F4R4N7	28.1	9.9	**6.0**	1	1	1	8.1
**Spot 23**	Cerato-platanin/ Barwin-related endoglucanase	JGI	*Pleurotus ostreatus*	PleosPC9_1 102212	14.8	5.2	**3.2**	1	1	1	10.5
	Aspartic protease	Uniprot	*Pholiota nameko*	G3XKT3	42.8	5.5	**3.9**	1	1	1	3.7
**Spot 24**	Aspartic-type endopeptidase activity	JGI	*Phlebia brevispora*	Phlbr1 115558	43.4	4.9	**2.9**	1	1	1	3.4
	Aspartic protease	Uniprot	*Pholiota nameko*	G3XKT3	42.8	5.5	**3.7**	1	1	1	3.7
**Spot 25**	Cerato-platanin/ Barwin-related endoglucanase	JGI	*Pleurotus ostreatus*	PleosPC9_1 102212	14.8	5.2	**3.1**	1	1	1	10.5
	Putative uncharacterized protein	Uniprot	*Puccinia graminis*	E3JYE0	21.2	6.3	**2.7**	1	1	1	6.4
**Spot 26**	Splicing coactivator SRm160/300	JGI	*Hydnomerulius pinastri*	Hydpi2 28112	79.5	10.4	**1.9**	1	1	1	1.9
	Aspartic protease	Uniprot	*Pholiota nameko*	G3XKT3	42.8	5.5	**3.9**	1	1	1	3.7
**Spot 27**	Serine-type peptidase activity/ subtilase activity	JGI	*Punctularia strigosozonata*	Punst1 106327	59.2	4.9	**53.1**	1	1	1	4.1
**Spot 28**	Cerato-platanin/ Barwin-like endoglucanases	JGI	*Pleurotus ostreatus*	PleosPC9_1 102212	14.8	5.2	**3.4**	1	1	1	10.5
	Putative uncharacterized protein hypP2	Uniprot	*Moniliophthora perniciosa*	Q6U7U4	47.9	8.7	**5.6**	1	1	1	4.7
**Spot 29**	α-N-arabinofuranosidase activity	JGI	*Galerina marginata*	Galma1 227157	66.6	8.0	**4.9**	1	1	1	1.6
	Cellulase	Uniprot	*Irpex lacteus*	Q9Y724	55.8	4.6	**9.1**	1	2	2	3.6
	Cellobiohydrolase	Uniprot	*Irpex lacteus*	Q75NB5	54.8	5.3	**8.9**	2	3	3	7.3
	Cellobiose dehydrogenase	Uniprot	*Irpex lacteus*	Q6AW20	82.1	5.5	**7.2**	1	3	3	4.1
**Spot 30**	Cellobiohydrolase	Uniprot	*Irpex lacteus*	Q75NB5	54.8	5.3	**2.7**	2	1	1	1.7
**Spot 31**	Esterase/lipase/thioesterase	JGI	*Phanerochaete chrysosporium*	Phchr1 7398	54.0	5.2	**8.1**	3	1	1	2.8
	Cellobiohydrolase	Uniprot	*Irpex lacteus*	Q75NB5	54.8	5.3	**12.9**	2	2	2	5.0
**Spot 32**	Catalytic activity/ Esterase/lipase/thioesterase	JGI	*Phanerochaete chrysosporium*	Phchr1 7398	54.0	5.2	**19.4**	3	1	1	2.8
	Cellobiohydrolase	Uniprot	*Irpex lacteus*	Q75NB5	54.8	5.3	**6.3**	2	2	2	4.6

Table S1. Continued

Homologous to proteins		Database	Species	Accession number	MM (kDa)	pI	Score	Proteins	UP	Peptides (%)	Coverage
**Spot 33**	1. 4-β cellobiohydrolase. GH6/ 'CAZy_ID 260597*	JGI	*Punctularia strigosozonata*	Punst1 89180	47.1	4.9	**5.3**	6	1	1	4.7
	Cellobiohydrolase II	Uniprot	*Irpex lacteus*	B2ZZ24	47.2	5.3	**14.2**	1	3	3	9.7
**Spot 34**	Cellobiohydrolase II	Uniprot	*Irpex lacteus*	B2ZZ24	47.2	5.3	**8.5**	1	1	1	4.7
**Spot 35**	Histone H4 (Fragment)	Uniprot	*Moniliophthora perniciosa*	E2LLY3	8.8	11.6	**5.1**	14	1	1	12.7
**Spot 36**	Cellobiohydrolase II	Uniprot	*Irpex lacteus*	B2ZZ24	47.2	5.3	**37.1**	4	6	6	18.8
**Spot 37**	Peptidase S41	JGI	*Bjerkandera adusta*	Bjead1_1 256345	74.1	5.6	**7.3**	5	2	2	4.3
	Endoglucanase	Uniprot	*Irpex lacteus*	Q5W7K4	42.2	4.9	**63.1**	1	3	3	11.0
	Cellobiohydrolase II	Uniprot	*Irpex lacteus*	B2ZZ24	47.2	5.3	**16.7**	3	4	4	13.7
**Differential spots in submerged cultures of *I. lacteus***											
**Spot 38**	GH3*	JGI	*Phlebiopsis gigantea*	Phlgi1 81135	99.0	5.1	**152.4**	23	3	7	6.2
	GH3	JGI	*Pisolithus microcarpus*	Pismi1 463669	78.0	4.7	**111.1**	13	2	4	4.6
	GH3*/ β-glucosidase*	JGI	*Coniophora puteana*	Conpu1 112748	95.6	4.9	**87.3**	14	1	4	4.5
	GH3*	JGI	*Laccaria bicolor*	Lacbi2 178737	80.9	5.2	**31.8**	5	1	2	3.0
	GH2*	JGI	*Bjerkandera adusta*	Bjead1_1 463744	103.5	5.4	**23.0**	7	1	3	4.7
	GH2	JGI	*Phlebiopsis gigantea*	Phlgi1 94774	104.0	4.9	**19.5**	7	1	3	4.6
	GH3	Uniprot	*Serpula lacrymans*	F8PMW3	78.3	4.7	**63.3**	8	2	4	4.4
	β-glucosidase	Uniprot	*Postia placenta*	B8P3C1	84.6	4.7	**47.1**	1	1	2	3.1
	Polyporopepsin	Uniprot	*Irpex lacteus*	P17576	35.0	4.7	**28.1**	1	3	3	17.4
	GH3	Uniprot	*Laccaria bicolor*	B0D734	80.9	5.2	**22.6**	7	3	4	6.1
**Spot 39**	Six-hairpin glycosidase-like	JGI	*Trametes versicolor*	Trave1 35444	74.4	5.8	**46.4**	4	2	2	6.1
	GH3*	JGI	*Phlebiopsis gigantea*	Phlgi1 81135	99.0	5.1	**10.0**	29	4	4	4.8
	Putative uncharacterized protein	Uniprot	*Moniliophthora perniciosa*	E2M3P0	13.2	4.7	**3.4**	1	1	1	13.0
**Spot 40**	candidate glyoxal oxidase*	JGI	*Punctularia strigosozonata*	Punst1 68820	59.6	5.2	**1.9**	1	1	1	3.4
**Spot 41**	Peptidase S8 and S53	JGI	*Punctularia strigosozonata*	Punst1 106327	59.2	4.9	**64.4**	1	1	1	4.1
	Polyporopepsin	Uniprot	*Irpex lacteus*	P17576	35.0	4.7	**25.6**	1	2	2	13.2
**Spot 42**	GH15/glucan 1.4-α-glucosidase	JGI	*Trametes versicolor*	Trave1 28580	60.9	5.4	**58.4**	1	1	1	2.1
	Six-hairpin glycosidase-like	JGI	*Trametes versicolor*	Trave1 35444	74.4	5.8	**9.9**	4	2	2	6.1
	Exo-β-(1→3)-galactanase	Uniprot	*Irpex lacteus*	B9ZZS1	16.8	47.8	**6.7**	1	4	4	19.0
	Putative uncharacterized protein	Uniprot	*Coprinopsis cinerea*	A8NY20	7.2	57.0	**5.7**	3	2	2	5.2
**Spot 43**	Endonuclease/exonuclease/phosphatase family	JGI	*Phanerochaete chrysosporium*	Phchr1 3383	39.0	8.6	**24.8**	4	2	2	5.6
	Mannose-6-phosphatase	Uniprot	*Phanerochaete chrysosporium*	Q281W3	38.4	6.6	**71.6**	2	4	4	8.2

Table S2. Functional classification of proteins from *I. lacteus* secretome growing on wheat straw. Proteins were identified from the LC-MS/MS data from the entire secretome (EPP), searching against the Basidiomycota database of Uniprot. Different groups are ordered from the highest to the lowest score in each functional group.GH= glycoside hydrolase family. **Identified as *Irpex lacteus* proteins.

Homologous to predicted proteins (Uniprot)	Uniprot ID	MM (kDa)	pI	Score	Proteins	Unique peptides	Peptides	Coverage (%)
**Glycoside hydrolases**								
Cellobiohydrolase II**	B2ZZ24	47.2	5.3	606.0	1	13	14	37.4
Cellulase**	Q9Y724	55.8	4.6	193.3	1	9	9	28.5
Endoglucanase**	Q5W7K4	42.2	4.9	118.3	1	3	3	11.0
Cellobiohydrolase**	Q75NB5	54.8	5.3	106.0	1	11	11	29.4
Exo-β-(1-->3)-galactanase**	B9ZZS1	47.8	6.7	99.8	1	8	8	32.4
Rhamnogalacturonan-hydrolase**	B6E8Y7	46.7	6.9	84.5	1	10	10	33.9
Endo-1.4-β-xylanase A	Q9HEZ1	43.5	5.4	57.7	2	3	3	15.7
GH3	F8NLG7	89.6	5.0	47.2	2	1	2	3.1
Cellulase**	Q9Y722	54.5	4.9	45.0	1	6	6	14.9
GH3	F8PMW3	78.3	4.7	36.5	2	2	3	4.3
CellobiohydrolaseII	A8CED8	47.3	5.1	19.4	1	2	3	8.4
GH74	A9CSH7	88.3	5.3	15.3	1	3	3	4.9
GH35	B0DSN5	119.6	6.0	11.5	1	2	2	2.2
GH61	D8PNG1	23.9	5.7	11.3	1	2	2	8.7
**Oxidoreductases**								
Melanin-decolorizing enzyme (MnP)	B3IWB3	38.3	5.1	142.1	1	10	11	41.5
Peroxidase cpop21 (DyP1)	P87212	53.9	5.0	122.2	1	8	8	26.9
Manganese peroxidase 3	H2D7E4	38.6	4.5	80.5	1	3	4	15.6
Cellobiose dehydrogenase**	Q6AW20	82.1	5.5	65.6	1	9	9	14.9
Copper radical oxidase	Q0ZKA4	108.3	4.7	49.7	1	2	2	2.8
Manganese peroxidase 1	Q2HWK0	37.8	4.5	19.6	1	2	3	14.0
**Esterases**								
Acetyl xylan esterase	H2E9	38.9	6.5	142.2	1	2	2	10.1
**Proteases**								
Polyporopepsin**	P17576	35.0	4.7	307.2	1	8	9	48.5
Aspartic protease	G3XKT3	42.8	5.5	35.6	1	3	4	8.3
Aspartic peptidase A1	B0CYE2	44.1	5.1	32.7	1	3	5	11.5
Family A1 protease	Q281W1	41.9	5.9	15.1	2	2	2	2.7
**Phosphatases**								
Mannose-6-phosphatase	Q281W3	38.4	6.6	21.3	1	3	3	5.9
**Other functions**								
Ribonuclease T2**	Q8LW55	41.8	5.1	30.9	1	2	2	6.8
Ubiquitin (Fragment)	Q01868	6.2	8.6	5.3	48	2	2	32.7
**Unknown functions**								
Putative uncharacterized protein	E2LEF2	19.0	5.9	30.8	2	2	2	13.8
Putative uncharacterized protein	D8PQ40	44.1	4.9	28.4	1	2	4	4.9
Putative uncharacterized protein	A8NY20	57.0	5.7	19.5	1	3	3	6.7
Putative uncharacterized protein	B8PLG1	59.0	5.0	12.7	1	2	3	5.2
Putative uncharacterized protein	B8P882	71.2	6.9	12.0	1	2	2	4.0
Putative uncharacterized protein	F8NRS6	55.4	5.1	7.8	2	1	2	3.8

Table S3. Functional classification of proteins from *I. lacteus* secretome growing on wheat straw. Proteins were identified from the LC-MS/MS data from the entire secretome (EPP), searching against the Basidiomycota database from JGI. Different groups are ordered from the highest to the lowest score in each functional group. GH= glycoside hydrolase family; CBM= carbohydrate-binding module. *Annotated protein.

Homologous to predicted proteins (JGI)	JGI ID	MM (kDa)	pI	Score	Proteins	Unique peptides	Peptides	Coverage (%)
**Glycoside hydrolases**								
GH6/ CBM	Phchr1 133052	48.4	5.3	293.0	2	4	4	10.7
GH2*	Phlgi1 94774	104.0	4.9	96.9	1	3	3	4.6
GH3*	Phlgi1 81135	99.0	5.1	81.9	1	4	6	5.9
GH10/ CBM	Phchr1 138345	43.5	5.4	79.5	1	3	3	15.7
GH3*/ β-glucosidase*	Conpu1 112748	95.6	4.9	77.4	1	2	4	4.9
GH35/ β-galactosidase	Gansp1 116588	108.8	5.1	69.4	1	3	3	3.5
Six-hairpin glycosidase-like	Hetan2 48511	54.4	5.4	31.7	5	2	2	6.8
GH10*	Bjead1 24950	40.2	6.5	43.6	1	2	3	9.4
CBM*	Phlgi1 31010	35.1	4.6	34.6	1	2	2	7.0
α-1.2-mannosidase	Phchr1 133585	90.5	4.9	31.1	2	2	2	3.0
GH92*	Phaca1 255063	91.9	4.9	31.1	1	2	2	3.2
GH35*/ β-galactosidase	Phlgi1 129018	108.7	5.2	27.4	2	2	2	1.5
GH5/ CBM/ CAZy_ID 259705*	Dicsq1 65561	41.0	4.8	27.3	1	2	2	5.9
GH43*/ CBM35*	Bjead1 424941	47.5	6.1	25.6	1	3	3	8.5
GH7*	PleosPC9 100231	47.9	4.5	25.2	13	2	2	5.3
GH5*	Bjead1 100935	43.7	6.1	24.5	1	3	3	11.1
GH74*/ CBM1*	Phlgi1 98770	87.0	5.0	22.0	1	2	2	3.1
GH7/ CBM	Phchr1 127029	54.9	5.3	21.9	1	2	2	4.1
GH7*/ CBM	Phaca1 264060	53.8	4.3	19.7	1	2	2	4.9
GH61*	Dicsq1 102981	22.9	5.7	18.3	3	2	2	10.1
GH5*	Trave1 33056	38.1	4.7	16.7	2	2	2	4.7
**Oxidorreductases**								
Manganese peroxidase 9 short*	Phlbr1 147108	38.3	4.6	105.4	1	4	4	16.4
Glyoxal oxidase*	Punst1 68820	59.6	5.2	18.2	1	2	2	5.9
**Esterases**								
Esterase/lipase/thioesterase	Phchr1 129015	30.5	6.7	188.5	1	2	2	13.2
Carboxylesterase. type B	Phchr1 7398	54.0	5.2	69.3	3	2	2	5.2
**Proteases**								
Peptidase S53	Punst1 106327	59.2	4.9	109.5	1	2	2	6.1
Peptidase A1*	Lacbi2 292906	44.1	5.1	47.3	3	2	5	11.5
Peptidase A1	Hebcy1 58903	44.4	5.2	43.7	1	2	5	11.5
Peptidase A1	Phlgi1 460435	40.4	5.0	34.6	1	2	2	3.1
Peptidase A1	Sphst1 264141	33.9	5.2	29.6	1	2	2	6.1
Peptidase S10/ serine carboxypeptidase	Bjead1 39510	70.6	4.9	27.8	1	1	2	4.6
Amidase signature enzyme	Hetan2 10776	50.0	5.0	16.9	13	1	2	4.1
Peptidase S53	PleosPC15 1077652	63.4	5.6	16.3	2	2	2	4.1
Amidase signature enzyme	Dacsp1 53351	46.5	4.7	15.9	1	1	2	4.8
Peptidase A1	Plicr1 701179	43.3	5.1	15.6	1	3	3	4.5
**Phosphatases**								
Histidine acid phosphatase	Wolco1 27937	49.5	5.9	7.8	1	2	2	5.2

Table S3. Continued

Homologous to predicted proteins (JGI)	JGI ID	MM (kDa)	pI	Score	Proteins	Unique peptides	Peptides	Coverage (%)
**Other functions**								
Ferritin/ribonucleotide reductase-like	Hetan2 431223	91.2	4.8	40.5	1	1	3	3.8
β-1.6-N-acetylglucosaminyltransferase	Phchr1 121730	89.5	4.6	82.2	1	3	3	4.9
Neutral/alkaline nonlysosomal ceramidase	Phchr1 3167	72.5	6.3	13.5	1	2	2	4.8
**Unknown functions**								
Putative uncharacterized protein	Bjead 424391	61.1	5.2	49.5	1	3	3	5.6
Putative uncharacterized protein	Phaca1 204873	96.1	5.1	35.2	1	2	4	5.8
Putative uncharacterized protein	Phaca1 247599	33.3	4.6	34.6	1	2	2	10.8
Hypothetical protein*	Cersu1 119820	71.2	4.8	28.8	1	1	2	4.6
Putative uncharacterized protein	Phchr1 3383	39.0	8.6	22.5	1	3	3	5.9
Hypothetical protein*	Cersu1 155413	71.3	4.7	14.0	1	2	2	3.1

Table S4. Functional classification of proteins from *I. lacteus* secretome growing on submerged cultures in CSS. Proteins were identified from the LC-MS/MS data from the entire secretome (EPP), searching against the Basidiomycota database of Uniprot. Different groups are ordered from the highest to the lowest score in each functional group.GH= glycoside hydrolase family. **Identified as *Irpex lacteus* proteins.

Homologous to predicted proteins (Uniprot)	Uniprot ID	MM (kDa)	pI	Score	Proteins	Unique peptides	Peptides	Coverage (%)
**Glycoside hydrolases**								
Exo-β-(1->3)-galactanase**	B9ZZS1	47.8	6.7	202.2	1	11	11	50.5
GH3	F8NLG7	89.6	5.0	130.3	2	1	2	3.1
GH3	F8PMW3	78.3	4.7	111.6	2	2	3	4.3
Cellobiohydrolase**	Q75NB5	54.8	5.3	54.4	1	6	6	11.3
Endoglucanase**	Q5W7K4	42.2	4.9	51.7	1	3	3	11.0
Rhamnogalacturonan-hydrolase**	B6E8Y7	46.7	6.9	41.3	1	5	5	13.1
GH35	B0DSN5	119.6	6.0	34.6	1	2	2	2.2
Cellobiohydrolase II**	B2ZZ24	47.2	5.3	32.7	1	4	4	12.6
Exo-beta-1.3-glucanase	Q53UH0	46.1	4.7	29.3	1	2	2	8.8
**Oxidoreductases**								
Peroxidase cpop21 (DyP1)	P87212	53.9	5.0	684.8	1	13	13	47.1
Melanin-decolorizing enzyme (MnP)	B3IWB3	38.3	5.1	368.6	1	11	12	41.8
Manganese peroxidase 3	H2D7E4	38.6	4.5	56.7	1	3	4	15.6
Cellobiose dehydrogenase**	Q6AW20	82.1	5.5	33.4	1	5	5	5.5
Copper radical oxidase	Q0ZKA4	108.3	4.7	27.0	1	2	2	2.8
Glyoxal oxidase (Fragment)	Q7LIJ2	56.0	5.6	25.5	3	2	2	5.5
Peroxidase 1 (DyP2)	B0BK71	54.9	5.5	10.5	1	2	2	3.1
**Proteases**								
Polyporopepsin**	P17576	35.0	4.7	4867.5	1	13	14	60.6
Aspartic protease	G3XKT3	42.8	5.5	63.7	1	3	4	8.3
Aspartic peptidase A1	B0CYE2	44.1	5.1	52.0	1	3	5	11.5
Family A1 protease	Q281W1	41.9	5.9	11.6	2	2	2	2.7
Subtilisin-like protease (Fragment)	Q6H8Q3	86.9	4.8	6.7	1	2	2	1.8
**Phosphatases**								
Mannose-6-phosphatase	Q281W3	38.4	6.6	87.5	1	3	3	5.9
**Other functions**								
Ribonuclease T2**	Q8LW55	41.8	5.1	137.4	1	4	4	14.4
**Unknown function**								
Putative uncharacterized protein	D8PJY8	56.5	4.7	59.6	1	1	2	7.3
Putative uncharacterized protein	D8PQ40	44.1	4.9	53.2	1	2	4	4.9
Putative uncharacterized protein	A8NY20	57.0	5.7	46.1	1	2	3	6.7
Putative uncharacterized protein	B8PLG1	59.0	5.0	29.8	1	2	3	5.2
Putative uncharacterized protein	F8NRS6	55.4	5.1	27.6	2	1	2	3.8
Putative uncharacterized protein	E2LEF2	19.0	5.9	19.2	2	2	2	13.8
Putative uncharacterized protein	B8P882	71.2	6.9	19.0	1	2	2	4.0

Table S5. Functional classification of proteins from *I. lacteus* secretome growing on submerged cultures in CSS. Proteins were identified from the LC-MS/MS data from the entire secretome (EPP), searching against the Basidiomycota database from JGI. Different groups are ordered from the highest to the lowest score in each functional group. GH= glycoside hydrolase family; CBM= carbohydrate-binding module. *Annotated protein.

Homologous to predicted proteins (JGI)	JGI ID	MM (kDa)	pI	Score	Proteins	Unique peptides	Peptides	Coverage (%)
**Glycoside hydrolases**								
GH15*/ Glucan 1.4-α-glucosidase	Trave1 28580	60.9	5.4	1093.7	1	2	2	9.0
GH15/ Six-hairpin glycosidase-like/CAZy_ID 263180*	Hetan2 44412	56.4	4.7	262.0	1	2	2	9.8
GH3*	Phlgi1 81135	99.0	5.1	203.9	1	4	6	5.9
GH2*	Phlgi1 94774	104.0	4.9	161.2	1	1	3	4.6
GH3	Serla 133022	95.7	5.0	136.5	1	2	3	6.6
GH2*/ Galactose-binding like/Predicted β-mannosidase	Bjead 463744	103.5	5.4	128.8	1	2	4	5.8
GH125*/ Six-hairpin glycosidase-like	Phlgi1 97503	55.8	5.2	88.8	1	4	4	9.9
GH35/ Beta-galactosidase	Gansp1 116588	108.8	5.1	85.3	1	3	3	3.5
GH3	Phlbr1 29514	100.1	5.0	58.4	1	2	3	5.3
Six-hairpin glycosidase-like	Schco2 1185226	56.5	4.7	47.0	1	1	2	7.3
GH92*	Bjead 365447	90.2	5.2	40.5	1	3	4	5.7
GH92*	Phaca1 255063	91.9	4.9	39.7	1	2	2	3.2
GH5*	Phlgi1 100064	46.3	5.3	37.9	1	2	3	11.1
GH92*	Phaca1 123038	90.2	4.8	37.9	1	2	3	4.2
Six-hairpin glycosidase-like	Bjead 149865	79.0	5.4	37.8	1	2	2	4.5
GH43*/ CBM35*	Bjead 424941	47.5	6.1	32.3	2	2	2	4.7
GH35*/ β-galactosidase	Phlgi1 129018	108.7	5.2	32.1	2	2	2	1.5
Amidase signature	Gymlu1 179802	58.8	4.8	31.9	2	2	4	6.5
GH5*	Bjead 100935	43.7	6.1	30.1	1	3	4	13.9
GH7/CBM	Phchr1 127029	54.9	5.3	22.3	1	2	2	4.1
GH31*	Phlgi1 127611	98.1	5.3	20.2	3	2	2	1.6
GH30/ β-glucocerebrosidase	Phchr1 9011	58.8	6.0	18.4	1	2	2	3.9
GH31/ Maltase glucoamylase and related hydrolases	Bjead 214996	20.1	8.7	17.6	3	2	2	5.5
GH35/ β-galactosidase	Galma1 275642	109.3	8.5	16.6	1	2	2	2.3
**Oxidoreductases**								
Glyoxal oxidase/ Coper radical oxidase*	Trave1 117805	59.9	5.5	53.3	2	2	2	5.4
Manganese peroxidase*	Trave1 112835	38.3	4.5	46.1	1	3	3	11.6
Glyoxal oxidase*	Punst1 68820	59.6	5.2	32.1	1	2	2	5.9
Glyoxal oxidase	Dicsq1 104366	59.7	5.4	28.4	1	2	2	4.0
**Esterases**								
Carboxylesterase. type B	Phchr1 7398	54.0	5.2	1157.1	3	2	2	5.2
Carbohydrate Esterase Family 15 protein*	Punst1 122520	42.0	4.8	19.0	1	1	2	6.7

Table S5. Continued

Homologous to predicted proteins (JGI)	JGI ID	MM (kDa)	pI	Score	Proteins	Unique peptides	Peptides	Coverage (%)
**Proteases**								
Peptidase S53	Punst1 106327	59.2	4.9	2983.0	1	2	2	6.1
Peptidase S10. serine carboxypeptidase	Bjead 157470	51.8	5.4	79.1	1	2	2	6.6
Peptidase A1	Phlgi1 460435	40.4	5.0	58.6	1	2	2	3.1
Peptidase A1	Hebcy1 58903	44.4	5.2	53.6	1	2	5	11.5
Peptidase A1*	Lacbi2 292906	44.1	5.1	53.5	3	2	5	11.5
Peptidase S10. serine carboxypeptidase	Bjead 39510	70.6	4.9	37.9	2	2	2	4.6
Peptidase A1	Sphst1 264141	33.9	5.2	25.6	1	2	2	6.1
Amidase signature enzyme	Bjead 41819	57.7	5.1	24.7	4	1	3	5.4
Peptidase A1	Bjead 33438	42.0	8.6	24.7	1	2	2	2.7
Amidase signature enzyme	Dacsp1 53351	46.5	4.7	22.2	1	1	3	6.6
Peptidase A1	Phchr1 40125	41.8	5.2	20.5	1	2	2	3.3
Peptidase A1	Plicr1 701179	43.3	5.1	13.0	1	3	3	4.5
**Other functions**								
β-1.6-N-acetylglucosaminyltransferase	Phchr1 121730	89.5	4.6	38.6	1	3	3	4.9
Flagellar basal body rod protein	PleosPC9 87860	42.4	5.1	34.6	1	3	3	7.7
Ferritin/ribonucleotide reductase-like	Hetan2 431223	91.2	4.8	11.7	1	2	2	2.6
**Unknown function**								
DUF1793	Trave1 35444	74.4	5.8	71.2	1	2	2	6.1
Putative uncharacterized protein	Phchr1 3383	39.0	8.6	68.7	1	3	3	5.9
Putative uncharacterized protein	Bjead 424391	61.1	5.2	27.3	1	3	3	5.6
Putative uncharacterized protein	Fompi1 128275	42.7	4.3	26.3	1	1	2	4.8
Hypothetical protein*	Cersu1 155413	71.3	4.7	23.0	1	2	2	3.1
Putative uncharacterized protein	Phaca1 247599	33.3	4.6	15.8	1	2	2	10.8
DUF1793	Punst1 132507	74.4	4.8	14.4	1	2	2	6.4
Putative uncharacterized protein	Sphst1 194507	29.2	5.8	5.2	2	2	2	6.0

Table S6. Functional classification of proteins from *P. chrysosporium* secretome growing on wheat straw. Proteins were identified from the LC-MS/MS data from the entire secretome (EPP), searching against the *P. chrysosporium* database of Uniprot. Different groups are ordered from the highest to the lowest score in each functional group.GH= glycoside hydrolase family; CBM= carbohydrate-binding module.

**Predicted proteins (Uniprot)**	**Uniprot ID**	**MM (kDa)**	**pI**	**Score**	**Proteins**	**Unique peptides**	**Peptides**	**Coverage (%)**
**Glycoside hydrolases**								
Glucan 1.3-β-glucosidase	Q2Z1W1	82.0	5.8	327.6	1	8	8	15.2
Cellulase	Q7LIJ0	53.8	4.9	286.6	2	13	13	35.7
Endo-1.4-β-xylanase A	Q9HEZ1	43.5	5.4	209.8	2	4	6	23.3
Cellobiohydrolase II (Fragment)	H3K419	46.3	5.1	189.6	2	6	6	20.1
Endo-1.4-β-xylanase C	B7SIW2	42.3	4.9	151.9	2	6	8	29.6
Exoglucanase 1	P13860	54.8	5.5	117.1	1	7	7	16.5
Endo-β-glucanase	C6H0M6	33.6	5.4	113.8	1	6	6	20.0
β-glucosidase (Fragment)	Q8TGC6	83.4	5.7	62.9	1	5	5	8.2
Mannan endo-1.4-beta-mannosidase activity/CBM	Q0PQY7	48.7	4.4	56.8	1	2	2	6.8
Galactan 1.3-β-galactosidase	Q50KB2	47.8	5.9	51.3	1	5	5	16.3
Mannan endo-1.4-beta-mannosidase activity/CBM	Q0PQY8	49.0	5.0	49.4	1	2	2	6.7
Endo-1.4-beta-xylanase B	B7SIW1	30.4	6.1	47.9	3	2	2	11.0
Endoglucanase	Q66NB7	40.4	5.3	41.5	1	3	3	17.6
α-galactosidase	Q9HFZ9	48.5	4.8	20.9	1	3	3	7.8
**Oxidoreductases**								
Manganese peroxidase 3	Q1K9D0	39.8	4.6	348.7	1	8	8	33.8
Copper radical oxidase	Q0ZKA8	67.8	5.5	265.8	1	11	11	26.9
Cellobiose dehydrogenase	Q12661	81.9	5.3	113.5	2	10	10	15.8
Glyoxal oxidase	Q01773	59.1	5.2	78.7	1	9	9	18.3
Lignin peroxidase isozyme H8 (Fragment)	D1M7B6	35.1	4.7	52.3	5	1	3	11.5
Ligninase LG5 (LiP)	P11543	39.4	4.6	45.0	1	2	4	13.2
Copper radical oxidase	Q0ZKA7	70.3	4.8	35.0	2	2	2	5.4
Manganese peroxidase 1 (Fragment)	Q6T246	20.3	4.6	30.1	4	2	2	15.6
Copper radical oxidase	Q0ZKA5	107.9	4.9	16.3	1	3	3	4.4
Pyranose 2-oxidase	Q6QWR1	69.3	6.5	14.3	1	2	2	2.9
1.4-benzoquinone reductase	Q9Y763	21.4	6.2	12.9	1	2	2	15.4
**Proteases**								
Family S53 protease	Q281W2	58.4	4.9	133.8	1	3	3	9.9
Family A1 protease	Q281W1	41.9	5.9	53.0	1	4	4	12.6
Subtilisin-like protease (Fragment)	Q6H8Q3	86.9	4.8	37.8	1	5	5	5.2
**Phosphatase enzymes**								
Mannose-6-phosphatase	Q281W3	38.4	6.6	85.3	1	9	9	30.5
**Other functions**								
Putative laminarinase	Q874E3	33.9	5.2	88.0	1	3	3	12.0
Actin (Fragment)	Q7Z8L2	39.5	5.7	19.6	1	4	4	14.0
Translation elongation factor EF1-alpha (Fragment)	Q5EG85	34.1	8.4	18.8	1	3	3	8.3
14-3-3 1 protein	Q562H7	28.8	4.9	10.2	1	2	2	6.7

Table S7. Functional classification of proteins from *P. chrysosporium* secretome growing on wheat straw. Proteins were identified from the LC-MS/MS data from the entire secretome (EPP), searching against the *P. chrysosporium* database of JGI. Different groups are ordered from the highest to the lowest score in each functional group. GH= glycoside hydrolase family; CBM= carbohydrate-binding module. *Annotated protein.

Predicted proteins (JGI)	JGI ID (Phchr1)	MM (kDa)	pI	Score	Proteins	Unique peptides	Peptides	Coverage (%)
**Glycoside hydrolases**								
GH10	125669	35.2	5.6	454.7	1	9	9	32.8
Glucan 1.3-β-glucosidase	8072	84.4	5.8	296.7	1	7	7	11.9
GH7/CBM	137372	58.1	5.2	284.5	1	11	13	33.0
GH28/ polygalacturonase	3805	39.8	5.3	239.5	1	8	8	29.1
GH10	7045	31.0	7.4	217.8	1	4	8	43.5
GH6/CBM	133052	48.4	5.3	189.6	1	6	6	19.1
GH10/CBM	138345	43.5	5.4	183.6	1	4	6	23.3
GH7/CBM	129072	53.9	4.8	155.8	2	5	8	23.4
GH155/Glucan 1.4-α-glucosidase	138813	60.8	5.4	153.7	1	10	10	26.1
GH10/CBM	138715	42.3	4.9	151.9	1	6	8	29.6
α-1.2. mannosidase	133585	90.5	4.9	147.3	1	11	11	17.2
CBM	3097	42.8	5.3	137.7	1	3	3	13.5
CBM	130517	49.2	5.9	137.0	1	4	6	17.0
GH18/ chitinase active site / CBM	6412	49.2	5.1	130.7	1	6	6	23.7
GH47	4550	59.5	4.9	122.0	1	10	10	23.6
GH18/ chitinase active site / CBM	134311	54.9	6.2	117.4	1	7	7	16.7
GH7/CBM	127029	54.9	5.3	117.1	2	4	7	16.5
GH/ BNR repeat	28013	66.6	4.8	107.0	3	7	8	18.1
GH13/α- amylase	38357	52.1	4.9	103.3	1	7	7	14.9
GH7/CBM	137216	53.8	4.5	102.4	2	2	5	14.7
GH10/CBM	139732	37.7	5.4	101.7	1	2	6	22.7
GH3	9257	83.8	5.7	94.1	1	11	11	17.4
Glucan 1.3-β-glucosidase	135724	46.5	5.9	92.3	1	8	8	32.0
Putative glucanase precursor	10833	33.3	5.2	88.0	1	3	3	12.2
GH/ chitinase active site	137237	30.4	6.8	88.0	1	4	4	20.0
GH35/ β-galactosidase	9466	113.3	6.0	87.4	1	8	8	9.5
GH5*	6433	58.0	4.5	85.4	1	5	5	17.9
GH30	9011	58.8	6.0	80.2	1	5	5	9.5
Endoglucanase-4	138739	33.6	5.5	78.9	1	3	3	15.7
GH71	134357	48.4	7.4	73.9	1	4	4	11.3
GH88	840	49.7	5.2	73.4	1	3	3	7.8
GH61/CBM	31049	34.5	5.4	71.5	1	2	2	11.0
GH18/ chitinase active site	39872	42.8	4.5	68.8	1	4	4	12.9
GH28	29397	44.3	5.5	67.8	1	5	5	12.0
GH5/CBM	4361	49.2	6.7	67.4	1	3	3	11.0
GH3/CBM	134658	85.4	5.7	62.9	1	5	5	8.0
GH3	36045	90.8	6.2	62.4	5	8	8	10.2
GH3	139063	88.0	5.4	60.3	1	8	8	8.6
GH12	7048	27.0	4.9	57.9	1	2	2	10.0

Table S7. Continued

**Predicted proteins (JGI)**	**JGI ID (Phchr1)**	**MM (kDa)**	**pI**	**Score**	**Proteins**	**Unique peptides**	**Peptides**	**Coverage (%)**
α-N-arabinofuranosidase A	3651	55.3	5.4	57.3	1	2	2	4.5
GH5/CBM	5115	40.8	4.5	56.8	1	2	2	8.2
GH5/CBM	140501	47.7	4.9	49.4	1	2	2	7.0
GH11/CBM	133788	30.4	6.1	47.9	1	2	2	11.0
GH13/starch-binding/ α-amylase	7087	61.1	5.6	47.8	1	4	4	9.0
Glucan 1.3-β-glucosidase	132568	76.5	6.2	47.7	1	3	3	5.2
GH18/chitinase active site	128098	45.3	4.2	47.1	1	2	2	5.3
β-mannosidase	135385	100.6	5.1	45.4	1	4	4	5.8
α-1.2.mannosidase	1930	93.1	5.2	43.7	1	4	4	6.4
GH31	125462	103.6	5.5	43.2	2	7	7	11.5
GH. clan GH-D	4422	30.3	4.7	43.1	1	4	4	14.7
GH43	4822	32.9	5.1	35.8	1	2	2	9.1
GH37	140627	72.7	4.8	32.7	1	4	4	6.6
GH. BNR repeat	138266	77.7	5.0	32.2	1	4	5	7.1
GH18/ chitinase active site	2991	48.9	6.5	32.2	1	3	3	14.3
GH61	41123	23.6	7.1	31.1	1	3	3	16.3
GH27. clan GH-D	125033	47.7	4.9	29.6	1	4	4	11.6
GH43	297	49.1	9.3	26.2	1	2	2	5.9
GH43	133070	33.8	5.4	24.2	1	3	3	12.6
GH3	129849	86.8	4.9	19.7	1	5	5	6.7
GH20	37522	46.9	5.1	18.9	1	2	2	5.5
Endo-1.4-β-galactosidase	138710	36.7	6.0	14.7	1	2	2	12.6
GH5	5773	74.2	5.0	12.7	1	4	4	7.1
GH28	140428	87.3	5.2	12.6	1	2	2	3.1
**Oxidoreductases**								
Haem peroxidase/ Fungal lignin peroxidase (MnP3)	878	39.9	4.6	342.0	2	7	8	33.8
Glyoxal oxidase	124009	67.8	5.5	262.2	1	10	10	23.6
GMC oxidoreductase/ Pyridine nucleotide-disulphide oxidoreductase	11098	82.0	5.5	113.5	1	10	10	15.8
Haem peroxidase/ Fungal lignin peroxidase (MnP2)	3589	40.1	4.7	105.7	2	1	2	4.5
Glyoxal oxidase	11068	59.1	5.3	69.4	1	8	8	16.6
Haem peroxidase/ Fungal lignin peroxidase (LiPA)	10957	39.6	4.6	50.2	4	1	3	10.2
Haem peroxidase/ Fungal lignin peroxidase (LiPC)	131738	39.3	4.6	43.0	4	2	4	13.2
Glyoxal oxidase	134241	80.8	5.1	35.0	1	2	2	4.7
FAD-dependent pyridine nucleotide-disulphide oxidoreductase	135167	51.1	6.9	30.4	2	4	4	9.8
Haem peroxidase/ Fungal lignin peroxidase (MnP1)	140708	39.5	5.0	30.1	2	2	2	7.9
Aldehyde dehydrogenase	8882	109.0	5.0	25.7	2	4	4	6.1
Aldehyde dehydrogenase*	137014	55.4	6.6	25.6	1	4	4	8.8
GMC oxidoreductase	6270	66.6	5.7	23.8	1	3	3	6.3
Aldo/keto reductase family proteins	7571	35.5	6.8	21.2	1	2	2	6.3
Zinc-containing alcohol dehydrogenase superfamily*	1675	40.4	6.1	15.6	1	2	2	5.3
FAD linked oxidase	3896	65.1	6.0	15.4	1	3	3	6.7
Flavodoxin/nitric oxide synthase*	10307	21.4	6.2	12.9	1	2	2	15.4
Haem peroxidase/ Fungal lignin peroxidase (LiP)	8895	39.4	4.7	11.3	2	1	2	8.1
D-isomer specific 2-hydroxyacid dehydrogenase. NAD-binding	140211	39.2	6.9	9.3	2	2	2	5.3
Aldo/keto reductase	125914	40.6	7.0	8.7	2	2	2	4.4
GMC oxidoreductase	126879	73.1	6.7	4.8	1	2	2	3.2
Alkyl hydroperoxide reductase. thiol specific antioxidant and related enzymes*	10009	25.0	6.3	4.6	1	2	2	9.5

Table S7. Continued

**Predicted proteins (JGI)**	**JGI ID (Phchr1)**	**MM (kDa)**	**pI**	**Score**	**Proteins**	**Unique peptides**	**Peptides**	**Coverage (%)**
**Esterases**								
Esterase/lipase/thioesterase	126075	35.6	6.4	81.2	2	3	3	15.1
Lipolytic enzyme. G-D-S-L	10607	41.2	5.6	54.0	1	4	4	10.7
Esterase/lipase/thioesterase	126191	56.0	5.5	49.1	1	6	6	14.2
Pectinesterase	132137	36.0	6.2	42.0	1	4	4	12.1
Esterase/lipase/thioesterase	7398	54.0	5.2	29.2	1	3	3	8.4
Esterase/lipase/thioesterase / Peptidase S28	37642	50.5	5.4	28.6	1	2	2	6.2
Phosphoesterase	30283	36.1	4.4	15.4	1	2	2	7.1
Esterase/lipase/thioesterase	3761	54.6	6.3	13.1	1	2	2	4.7
Pectinesterase	8580	45.3	5.9	9.2	1	3	3	6.7
**Proteases**								
Peptidase S8 and S53. subtilisin. kexin. sedolisin	26825	59.1	5.2	219.0	1	6	6	22.7
Peptidase aspartic. active site/ Peptidase A1. pepsin	8470	44.2	5.0	194.1	1	4	5	20.2
Peptidase aspartic. active site/ Peptidase A1. pepsin	8469	44.8	5.0	187.1	1	6	7	19.9
Amidase	3346	55.2	5.5	136.3	1	9	9	21.1
Peptidase S10. serine carboxypeptidase	8913	97.3	8.5	134.7	2	4	4	6.2
Tripeptidyl aminopeptidase	133020	59.0	4.9	133.8	1	3	3	9.8
Peptidase S10. serine carboxypeptidase	3855	51.6	5.4	100.1	1	4	4	14.9
Peptidase A4. scytalidopepsin B	43144	21.7	4.5	93.8	2	2	5	29.9
Peptidase aspartic. active site/ Peptidase A1	138924	41.2	5.7	93.0	1	6	6	18.6
Peptidase aspartic. active site/ Peptidase A1	129956	38.6	4.6	92.1	1	5	5	14.9
Peptidase A4. scytalidopepsin B/ Aspartic-type endopeptidase activity	121400	27.2	5.2	77.9	1	2	3	19.4
Peptidase aspartic. active site/ Peptidase A1	128676	39.6	4.6	67.7	2	2	2	6.8
Peptidase aspartic. active site/ Peptidase A1	29558	33.4	4.4	67.1	1	2	2	9.2
Peptidase aspartic. active site/ Peptidase A1	135608	41.9	5.9	53.0	1	4	4	12.6
Peptidase A4. scytalidopepsin B/ Aspartic-type endopeptidase activity	120995	26.8	6.2	51.6	1	1	3	9.4
Peptidase A4. scytalidopepsin B/ Aspartic-type endopeptidase activity	8468	44.2	5.4	48.0	1	3	3	7.9
Peptidase aspartic. active site/ Peptidase A1	8008	42.9	6.6	41.6	2	3	3	8.1
Protease-associated PA/ Peptidase S8 and S53. subtilisin. kexin. sedolisin	133799	93.3	5.0	37.8	1	5	5	4.8
Peptidase A4. scytalidopepsin B	7318	28.6	4.5	31.9	1	3	3	18.1
Amidase	140657	68.8	6.5	22.9	1	3	3	7.1
Protease-associated PA/ Peptidase S8 and S53. subtilisin. kexin. sedolisin	133613	87.5	5.4	20.4	1	3	3	3.7
Peptidase S8 and S53. subtilisin. kexin. sedolisin	1483	61.1	5.9	18.5	1	2	2	3.9
Peptidase S8 and S53. subtilisin. kexin. sedolisin	129261	64.1	5.9	15.8	1	2	2	3.7
Amidase	3719	55.1	5.4	14.2	1	2	2	3.5
Peptidase aspartic. active site/ Peptidase A1	126189	36.9	4.2	14.1	1	2	2	6.4
Peptidase S10. serine carboxypeptidase	2656	67.9	5.5	14.1	2	3	3	4.9
Peptidase. eukaryotic cysteine peptidase active site	123502	70.6	5.2	11.5	2	2	2	5.9
Peptidase aspartic. active site/ Peptidase A1	138453	33.5	4.2	10.5	1	2	2	6.1
Peptidase aspartic. active site/ Peptidase A1	7917	44.9	6.2	9.4	1	2	2	4.0
Peptidase aspartic. active site/ Peptidase A1	131827	34.9	4.6	4.7	1	2	2	7.2
Peptidase A1. pepsin	8365	62.4	4.9	4.2	1	2	2	2.5
**Phosphatase enzymes**								
Histidine acid phosphatase	137138	60.4	6.2	282.1	2	9	9	17.8
Endonuclease/exonuclease/phosphatase	138954	64.8	5.9	55.8	1	1	3	4.9
**Other functions**								
Aminotransferase	138738	65.4	4.7	156.6	1	10	12	24.5
Lipocalin-related protein and Bos/Can/Equ allergen	123909	33.8	5.5	109.3	1	6	6	19.9
Survival protein SurE	5655	31.5	4.7	92.7	1	4	4	12.5
Polysaccharide deacetylase	124827	37.5	4.8	80.2	1	4	4	17.4

Table S7. Continued

Predicted proteins (JGI)	JGI ID (Phchr1)	MM (kDa)	pI	Score	Proteins	Unique peptides	Peptides	Coverage (%)
Pectin lyase-like	8645	23.0	6.0	76.9	1	6	6	24.6
Thaumatin. pathogenesis-related	3280	26.8	5.0	66.3	1	1	2	12.9
Thaumatin. pathogenesis-related	5297	28.4	5.3	62.4	1	1	2	12.3
Ribonuclease T2	126123	41.4	5.2	43.3	1	3	3	11.8
Protein prenyltransferase	6458	39.6	5.3	41.5	1	3	3	18.0
Polysaccharide lyase family 8	6736	92.3	4.8	39.2	1	3	3	4.7
Porin. eukaryotic type	138775	31.4	8.9	38.2	1	5	5	20.1
Glutathione S-transferase	140259	25.4	7.1	30.1	1	5	5	18.0
Parallel beta-helix repeat	1249	51.1	5.4	29.4	1	3	3	6.8
Polysaccharide deacetylase/ tubulin	132376	54.5	4.8	23.3	1	3	3	7.0
Stomatin	40896	29.7	8.1	20.3	1	3	3	11.0
Actin/actin-like	139298	52.5	6.7	19.6	1	4	4	10.6
Glycosyl transferase. family 35	5751	98.2	6.4	19.1	1	3	3	3.1
Translation elongation factor EF-1/ Protein synthesis factor. GTP-binding*	134660	50.1	9.1	16.8	1	3	3	5.7
Glycosyl transferase. group 1	122462	81.6	6.5	15.1	1	2	2	2.9
Cys/Met metabolism pyridoxal-phosphate-dependent enzymes	10555	45.6	6.8	14.4	2	2	2	5.4
N-6 Adenine-specific DNA methylase	137275	69.3	6.5	14.3	1	2	2	2.9
14-3-3 protein (putative ortholog to S. cerevisiae Protein BMH2*)	139500	27.3	5.1	10.2	1	2	2	7.1
Proteasome α-subunit	133187	29.4	7.5	9.7	1	2	2	8.2
E1 protein and Def2/Der2 allergen	6572	18.3	4.9	4.7	1	2	2	16.1
**Unknown function**								
Putative uncharacterized protein	130748	57.6	4.8	295.7	1	2	2	6.7
Putative uncharacterized protein	964	32.8	5.6	193.2	1	7	7	28.1
Putative uncharacterized protein	140079	75.3	5.9	186.7	1	10	10	22.7
Putative uncharacterized protein	5607	48.9	5.7	132.5	1	6	6	23.1
Putative uncharacterized protein	8738	11.9	7.1	102.8	3	4	5	75.7
Putative uncharacterized protein	6991	51.3	4.7	87.3	1	7	7	23.7
Putative uncharacterized protein	3383	39.0	8.6	85.3	1	9	9	30.3
Putative uncharacterized protein	6482	44.3	5.0	65.7	1	4	6	17.2
Putative uncharacterized protein	3328	78.4	6.1	59.6	1	7	7	10.2
Putative uncharacterized protein	6450	83.1	5.0	57.4	1	5	5	8.5
Putative uncharacterized protein	6069	65.7	4.9	47.9	1	4	4	9.4
Putative uncharacterized protein	7029	37.1	5.2	46.4	1	4	4	13.2
Putative uncharacterized protein	7437	47.1	7.0	46.1	1	4	4	10.5
Putative uncharacterized protein	3431	67.6	4.8	43.2	1	4	4	8.5
Uncharacterised conserved protein UCP028846	139777	53.8	5.6	41.9	1	6	6	11.6
Putative uncharacterized protein	123916	37.0	4.9	41.1	1	4	4	12.1
Putative uncharacterized protein	122884	73.0	5.3	40.5	1	4	4	7.7
Putative uncharacterized protein	3168	49.8	6.5	39.0	1	5	5	13.4
Putative uncharacterized protein	2035	28.8	4.4	36.1	1	3	3	17.1
Protein DUF338*	138982	58.7	4.7	30.3	1	4	4	11.7
Putative uncharacterized protein	5774	59.6	5.2	26.6	1	3	3	6.0
Putative uncharacterized protein	125335	68.0	5.7	26.1	1	2	2	3.9
Putative uncharacterized protein	3653	42.2	5.0	24.8	1	2	2	6.1
Putative uncharacterized protein	4912	76.8	10.5	23.9	1	2	2	4.2
Putative uncharacterized protein	8737	11.5	5.9	23.7	3	1	2	24.8
Protein DUF338	7122	57.5	4.5	22.3	1	3	3	8.3
Putative uncharacterized protein	2256	72.3	5.6	22.2	1	4	4	5.9

Table S7. Continued

**Predicted proteins (JGI)**	**JGI ID (Phchr1)**	**MM (kDa)**	**pI**	**Score**	**Proteins**	**Unique peptides**	**Peptides**	**Coverage (%)**
Putative uncharacterized protein	122292	66.2	5.8	19.2	1	3	3	5.4
Putative uncharacterized protein	6997	83.8	5.7	14.2	1	2	2	3.8
Putative uncharacterized protein	134621	33.1	4.5	14.2	2	2	2	9.9
Putative uncharacterized protein	1999	63.5	5.2	12.0	1	2	2	3.9
Putative uncharacterized protein	2037	30.4	5.5	11.2	1	2	2	10.8
Putative uncharacterized protein	2163	15.2	6.3	10.9	1	2	2	15.7
Putative uncharacterized protein	7809	45.7	6.4	9.1	1	2	2	4.2
Putative uncharacterized protein	280	101.0	5.1	7.6	1	2	2	2.5
Putative uncharacterized protein	4690	79.3	7.1	5.6	1	2	2	4.1

Table S8. Functional classification of proteins from *P. ostreatus* secretome growing on wheat straw. Proteins were identified from the LC-MS/MS data from the entire secretome (EPP), searching against the *P. ostreatus* database of Uniprot. Different groups are ordered from the highest to the lowest score in each functional group.

**Predicted proteins (Uniprot)**	**Uniprot ID**	**MM (kDa)**	**pI**	**Score**	**Proteins**	**Unique peptides**	**Peptides**	**Coverage (%)**
**Glycoside hydrolases**								
α-L-arabinofuranosidase	G0TES6	68.9	8.1	52.2	1	3	3	6.4
Cellulose 1.4-β-cellobiosidase	A5AA53	49.3	5.6	50.6	1	4	4	9.4
Cellulose 1.4-β-cellobiosidase	A5AA50	55.6	5.0	37.0	3	2	2	3.4
**Oxidoreductases**								
Versatile peroxidase 2	G8FPZ2	38.5	4.7	629.7	3	8	8	35.8
Laccase	Q96TR4	57.4	6.1	284.1	1	9	9	20.9
Manganese peroxidase 3	O74179	36.9	4.8	24.4	3	2	2	4.8
Laccase	D4AIA5	56.1	6.8	11.4	3	3	3	7.4
Glyceraldehyde-3-phosphate dehydrogenase	D0VBH9	36.0	7.2	10.7	4	2	2	6.3
**Proteases**								
Subtilisin-like protease	Q6ZYK6	93.2	5.3	330.3	2	11	11	21.1
Peptidyl-Lys metalloendopeptidase	P81055	17.9	6.2	118.5	1	5	5	46.4
Putative aspartyl-proteinase (Fragment)	Q96TV7	18.5	6.2	51.8	1	3	3	21.4
Peptidase 1	C4PFY6	38.7	8.2	42.4	1	2	2	13.0
**Other functions**								
Ribonuclease T2	Q75NB1	41.5	6.4	65.1	1	4	4	14.2
Bilirubin oxidase	Q9UVY4	56.8	4.9	33.3	7	3	3	9.9
Putative ubiquitin (Fragment)	Q96TW1	27.3	8.9	28.3	1	3	3	33.3
**Unknown function**								
Putative uncharacterized protein	D2JY75	27.8	6.6	162.8	2	7	7	42.8
Putative uncharacterized protein	D2JY77	26.4	10.0	6.4	1	2	2	7.9

Table S9. Functional classification of proteins from *P. ostreatus* secretome growing on wheat straw. Proteins were identified from the LC-MS/MS data from the entire secretome (EPP), searching against the *P. ostreatus* database of JGI. Different groups are ordered from the highest to the lowest score in each functional group.GH= glycoside hydrolase family; CBM= carbohydrate-binding module. *Annotated protein.

**Predicted proteins (JGI)**	**JGI ID (PleosPC15)**	**MM (kDa)**	**pI**	**Score**	**Proteins**	**Unique peptides**	**Peptides**	**Coverage (%)**
**Glycoside hydrolases**								
GH7/ CBM	1092970	55.9	4.8	981.9	1	10	22	61.2
GH47	1053206	58.7	5.2	699.4	1	10	13	35.9
GH47	1104326	60.5	5.5	602.8	1	9	9	22.0
GH7/ Concanavalin A-like lectin/ Glucanase	1039666	52.8	5.1	503.4	1	6	18	48.1
GH3	1035754	82.8	6.0	295.2	1	13	13	23.8
GH47*	1035282	58.8	5.2	251.2	1	4	7	17.9
GH28*	39721	41.7	5.8	244.5	1	5	5	11.5
GH79*	62138	55.8	5.9	227.8	1	11	11	28.3
GH3*	41613	76.5	5.9	209.5	1	10	11	17.5
GH5*/ CBM	1067505	40.9	5.1	176.3	1	9	9	40.8
GH6*/ CBM	48333	47.2	5.3	169.9	1	7	7	20.7
GH7*	1038048	48.8	4.6	161.7	1	8	10	24.3
GH16*/ Concanavalin A-like lectin/ Glucanase	1040267	34.9	4.8	161.4	1	7	7	31.8
GH37* / Six-hairpin glycosidase-like	1046178	72.6	5.4	141.0	1	7	7	15.4
GH35*	1066752	113.2	6.5	134.0	1	14	14	17.5
GH72*	1062416	58.3	4.7	112.1	1	4	4	9.3
GH10*	1078540	35.7	6.8	93.1	1	6	6	21.5
GH15*/ Glucan 1.4-alpha-glucosidase	29106	61.4	5.4	84.4	1	5	5	16.4
GH2*	61779	106.7	5.6	73.4	1	7	7	8.5
GH76*/ Six-hairpin glycosidase-like	1064904	38.3	6.2	68.2	1	4	4	16.3
GH5*/ CBM	1101955	41.8	5.1	68.1	1	4	4	24.0
GH115*	1045181	16.4	9.6	65.9	1	4	4	30.9
GH115*	162095	109.5	5.4	64.4	1	10	10	13.1
GH12*	50765	26.1	5.8	62.0	1	4	4	25.3
GH3*	1049518	83.6	5.7	38.8	1	2	2	3.5
GH35*	1113377	82.2	6.4	38.4	1	5	5	8.1
GH31*	1061735	106.4	6.1	32.1	1	4	4	4.0
GH20*	1094009	59.5	6.3	30.3	1	3	3	8.7
GH	1036297	77.0	6.5	27.6	1	1	3	6.3
GH1	153105	69.1	6.5	26.1	1	3	3	4.8
α-L-rhamnosidase	1108884	41.2	7.6	26.0	1	2	2	9.5
GH20*/ GH-D	1064686	64.3	5.9	21.1	1	4	4	10.9
GH27*/ GH-D	51341	42.7	6.9	20.6	1	2	2	7.4
GH13*/ Carbohydrate-binding-like fold/ α-amylase	1095839	61.7	5.4	20.4	1	3	3	4.9
GH55*	37178	82.8	6.0	18.5	1	3	3	5.9
GH51*/ α-L-arabinofuranosidase*	1042120	65.0	9.3	16.9	1	2	2	4.3
GH88*/ GH105*	154213	44.7	6.8	16.8	1	2	2	4.5
GH5*	49423	46.2	6.7	16.2	1	2	2	6.1
GH105*	1063776	41.7	5.3	16.0	1	2	2	5.2
GH27*/ GH-D	1035175	43.6	5.0	15.8	1	2	2	8.0
GH3*	1074900	164.0	6.3	15.5	1	1	2	1.1

Table S9. Continued

Predicted proteins (JGI)	JGI ID (PleosPC15)	MM (kDa)	pI	Score	Proteins	Unique peptides	Peptides	Coverage (%)
Six-hairpin glycosidase-like	159035	41.6	7.9	13.2	1	2	2	6.5
GH92*/ Six-hairpin glycosidase-like	1110177	87.8	6.3	12.3	1	3	3	6.1
GH88*/ Six-hairpin glycosidase-like	1031712	43.6	8.2	11.7	1	2	2	5.3
GH35*/ Glycogen/starch/alpha-glucan phosphorylase	1058949	97.9	6.3	11.6	1	3	3	3.5
GH78*/ α-L-rhamnosidase/ Six-hairpin glycosidase-like	13903	65.7	5.7	10.1	1	2	2	4.3
**Oxidoreductases**								
Haem peroxidase/ Fungal lignin peroxidase (VP2)	199491	38.3	4.7	436.1	1	4	5	14.8
Multi-copper-oxidase Laccase2/ Similar to POXA3*	1067328	57.4	6.1	282.1	1	9	9	20.9
Manganese peroxidase 2*	199510	37.9	5.2	194.0	1	3	4	10.3
Short-chain dehydrogenase/reductase SDR/ NAD(P)-binding / Glucose/ribitol dehydrogenase	1114616	26.0	9.2	133.7	1	8	8	32.8
Short-chain dehydrogenase/reductase SDR / Glucose/ribitol dehydrogenase/ NAD(P)-binding	1069103	28.2	7.6	115.2	1	8	8	36.2
Flavodoxin/nitric oxide synthase / Flavoprotein WrbA	41896	21.5	6.5	106.8	1	4	4	32.2
Manganese peroxidase 5*	199511	37.7	4.7	91.5	1	5	5	17.5
Glyoxal oxidase / Galactose oxidase	1065295	59.6	5.7	89.1	1	5	5	13.0
Glyoxal oxidase	1078518	58.7	6.9	77.6	1	9	9	21.3
Aldehyde dehydrogenase	1090768	54.6	6.4	63.1	1	6	6	14.2
Glyoxal oxidase	1109338	61.7	6.8	61.4	1	7	7	16.6
Short-chain dehydrogenase/reductase SDR / NAD(P)-binding / Glucose/ribitol dehydrogenase	1033771	28.9	7.9	49.0	1	5	5	22.0
Cupin 2. conserved barrel / Bicupin. oxalate decarboxylase/oxidase	1078793	48.5	4.7	29.3	1	2	2	5.3
Manganese peroxidase 6*	1041740	38.1	5.1	24.5	1	3	3	10.0
Aldo/keto reductase	1102061	35.2	6.3	24.1	1	5	5	12.6
Manganese peroxidase 3*	1089546	37.3	4.8	22.5	1	2	2	4.8
Aldo/keto reductase	1075590	39.9	7.1	16.8	1	2	2	5.6
Short-chain dehydrogenase/reductase SDR / Glucose ribitol dehydrogenase	162527	26.3	6.0	15.4	1	2	2	10.4
Haem peroxidase. animal/ Cytochrome P450	1065994	111.6	6.5	13.1	1	2	2	2.4
6-phosphogluconate dehydrogenase. NAD-binding	1037028	26.6	7.5	10.1	1	2	2	9.4
Multicopper oxidase. type 1.2.3/ Laccase*	1113032	57.9	6.8	9.4	1	2	2	4.5
Aldo/keto reductase	1090974	37.1	5.9	8.3	1	3	3	7.7
Short-chain dehydrogenase/reductase SDR / Glucose/ribitol dehydrogenase/ NAD(P)-binding	1090751	31.0	8.4	5.6	1	2	2	10.2
**Esterases**								
Carboxylesterase. type B	1091241	59.3	7.0	1157.1	1	17	17	45.2
Carboxylesterase. type B	1047807	56.3	7.4	418.3	1	14	14	33.2
Lipase. GDSL / Esterase. SGNH hydrolase-type	1102068	26.5	7.5	129.1	1	7	7	44.9
Carboxylesterase. type B	1040351	56.7	8.5	110.8	1	8	8	18.0
Carboxylesterase. type B / C-type lectin fold	33340	76.1	6.1	82.6	1	7	7	12.9
Carboxylesterase. type B	1078816	54.4	7.6	51.7	1	7	7	21.0
Pectinesterase/ Carbohydrate Esterase Family 8 protein*	1044335	42.2	5.0	50.8	1	3	3	11.0
Carbohydrate Esterase Family 1*	1114413	38.9	6.2	38.7	1	2	2	6.0
Carbohydrate Esterase Family 15*	1086797	46.5	7.7	28.3	1	2	2	7.9
Glycerophosphoryl diester phosphodiesterase	1074827	39.9	6.9	24.2	1	2	2	5.5
Pectinesterase/ Carbohydrate Esterase Family 8*	1061918	35.6	8.6	20.0	1	3	3	17.7
Lipase. GDSL	1078000	45.6	6.0	17.5	1	3	3	8.1
Lipase. class 3	1044280	31.9	6.9	16.0	1	2	2	7.6
Carbohydrate Esterase Family 4*	1111329	48.4	4.8	13.3	1	2	2	5.1
Metallophosphoesterase	157761	112.6	5.1	12.9	1	2	2	1.8
Esterase. SGNH hydrolase-type/ Carbohydrate Esterase Family 16*	1075485	17.6	5.5	10.1	1	2	2	17.9
Carboxylesterase. type B/ Carboxylesterase and related proteins*	1051283	48.7	8.2	10.1	1	2	2	5.3
**Proteases**								
Peptidase S8 and S53	1077652	63.4	5.6	4612.3	1	10	10	24.3
Peptidase aspartic	1064571	37.5	6.2	2037.5	1	6	6	26.2

Table S9. Continued

Predicted proteins (JGI)	JGI ID (PleosPC15)	MM (kDa)	pI	Score	Proteins	Unique peptides	Peptides	Coverage (%)
Peptidase aspartic/ Similar to Merops A01A peptidase*	1040870	42.2	5.7	438.8	1	5	5	17.4
Peptidase aspartic. catalytic/ Similar to Merops A10A protease*	26137	32.1	5.0	369.4	1	5	5	38.7
Amidase signature enzyme	1046424	43.0	5.0	337.1	1	6	8	26.5
Peptidase S10. serine carboxypeptidase	175915	57.8	5.1	323.6	1	9	9	25.8
Peptidase S8 and S53	1112600	93.3	5.3	312.3	1	10	10	20.0
Peptidase S8 and S53	1039782	62.6	5.8	287.9	1	5	5	8.3
Peptidase S10/ Serine carboxypeptidase	1073281	49.5	4.7	260.0	1	8	8	31.5
Peptidase S41	1111045	74.2	6.0	215.8	1	12	12	21.3
Peptidase M36	62198	62.9	6.1	209.7	1	5	5	11.9
Amidase signature enzyme	1032663	32.7	5.1	181.0	1	5	9	28.9
Metalloprotease	1037634	33.9	5.7	116.6	1	5	5	24.4
Amidase signature enzyme	20196	51.9	5.2	100.0	1	2	5	8.9
Peptidase A1/ Peptidase aspartic*	1055405	43.3	5.1		1	5	5	14.6
Peptidase S10/ Serine carboxypeptidase	1078405	51.7	5.8	74.4	1	6	6	19.0
Peptidase S8 and S53	1088548	38.7	8.2	40.1	1	2	2	13.0
Peptidase M28	1079957	48.8	5.7	39.3	1	2	2	5.8
Peptidase C19/	1031730	26.2	4.6	38.4	1	3	3	19.8
Peptidase S41	1060558	70.8	5.3	30.3	1	5	5	9.4
Amine oxidase	1064593	71.1	6.3	29.1	1	2	2	4.6
Peptidase	10257	42.4	5.3	27.7	1	2	2	5.1
Peptidase A1/ Peptidase aspartic*	1089322	59.9	7.0	24.9	1	3	3	7.5
Peptidase S28	1045574	60.6	5.2	20.6	1	2	2	5.3
Protease-associated PA/ ABC transporter-like	157203	93.6	6.9	13.1	1	3	3	4.8
**Phosphatases**								
Phosphatase	1095212	34.7	5.5	167.0	1	8	8	33.1
Survival protein SurE-like phosphatase	162185	31.4	4.8	108.3	1	4	4	18.7
Histidine acid phosphatase	1110233	41.1	5.4	98.2	1	3	3	11.4
Histidine acid phosphatase	185948	47.7	6.5	51.8	1	4	4	10.6
Histidine acid phosphatase	1101887	60.7	6.2	34.0	1	4	4	8.0
Alkaline phosphatase	1099298	59.6	6.5	20.6	1	2	2	4.4
Histidine acid phosphatase	1035741	49.7	6.5	10.2	1	2	2	6.0
**Other functions**								
Thaumatin. pathogenesis-related	1081316	26.6	4.4	577.3	1	3	3	27.0
β-1.6-N-acetylglucosaminyltransferase. contains WSC domain	1114640	109.8	4.9	566.2	1	11	14	21.6
β-1.6-N-acetylglucosaminyltransferase. contains WSC domain	1081617	80.3	5.1	544.0	1	9	12	28.1
Phosphatidylserine decarboxylase-related	1062065	49.6	4.9	241.0	1	9	9	25.0
Site-specific DNA-methyltransferase (cytosine-N4-specific)*	1045048	33.0	7.0	231.0	1	6	6	25.9
Membrane attack complex component/perforin/complement C9	1086943	102.7	5.5	223.7	1	15	15	19.9
Amidohydrolase 2	1049583	19.7	4.5	139.9	1	2	2	11.5
α/β hydrolase fold-1	3428	29.9	6.7	137.3	1	5	5	22.9
Cerato-platanin / Barwin-related endoglucanase	1088025	14.4	7.7	136.0	1	2	2	18.9
NmrA-like/ NAD(P)-binding/ Polysaccharide Lyase Family1*	53399	31.8	6.4	69.5	1	5	5	19.9
Pectate lyase/Amb allergen/ Polysaccharide Lyase Family1*	1054721	32.5	6.8	68.4	1	5	5	23.6
Fumarylacetoacetase	1032901	33.1	7.0	62.5	1	6	6	15.1
Macrophage migration inhibitory factor	21661	13.3	7.2	50.3	1	3	3	27.5
Polysaccharide lyase family 8*	1101738	81.0	5.4	44.8	1	2	4	7.0
Ribonuclease T2	185836	40.8	6.3	44.7	1	3	3	9.6

Table S9. Continued

Predicted proteins (JGI)	JGI ID (PleosPC15)	MM (kDa)	pI	Score	Proteins	Unique peptides	Peptides	Coverage (%)
Transketolase	1113999	69.3	6.1	44.3	1	7	7	14.8
Hemopexin	1113759	26.2	5.1	42.2	1	5	5	25.3
Neutral/ Alkaline nonlysosomal ceramidase	1065698	69.4	6.9	34.4	1	5	5	7.5
SMP-30/ Gluconolaconase/ LRE-like region	169186	42.5	6.7	33.9	1	3	3	6.3
Ferritin/ Ribonucleotide reductase-like	1096735	44.8	5.7	26.9	1	4	4	11.5
Dienelactone hydrolase	1088515	30.3	5.9	25.0	1	2	2	10.3
Ubiquitin	1088454	42.7	8.1	24.4	6	2	2	23.6
YD repeat	1114460	218.4	6.2	23.5	1	3	3	1.6
Acetamidase/Formamidase	52279	43.0	5.5	23.4	1	2	2	7.0
Ribosomal protein L18e	1063630	20.9	11.3	14.2	1	3	3	20.4
Thaumatin. pathogenesis-related	21503	26.9	4.8	14.1	1	2	2	11.4
Thiolase/ Ribosomal protein S3. C-terminal	1062741	72.3	8.3	13.8	1	2	2	2.8
SMP-30/ Gluconolaconase/ LRE-like region	1109731	40.9	6.2	10.1	1	2	2	5.3
SMP-30/ Gluconolaconase/ LRE-like region	1042025	78.1	5.2	10.1	1	2	2	3.3
Amidohydrolase 2	53363	35.2	4.8	8.6	1	2	2	5.4
Ribosomal protein S4	1054562	22.3	10.7	8.6	1	2	2	7.8
Membrane attack complex component/perforin/complement C9	163136	55.0	5.3	4.9	1	2	2	4.7
Ribosomal protein S3Ae	1089059	29.5	9.9	4.4	1	2	2	7.0
Homogentisate 1.2-dioxygenase / Cupin. RmlC-type	1107843	53.1	6.2	4.0	1	2	2	3.6
**Unknown function**								
Putative uncharacterized protein	1079640	19.0	4.8	399.8	1	2	2	16.6
Putative uncharacterized protein	1080147	37.9	7.4	273.4	1	1	3	10.2
Putative uncharacterized protein	1080163	37.7	5.6	244.9	1	5	7	38.5
Putative uncharacterized protein	1066218	47.8	4.9	122.0	1	6	6	17.9
Putative uncharacterized protein	1032791	51.4	5.8	91.3	1	5	5	13.8
Putative uncharacterized protein	1076067	43.9	6.4	89.3	1	5	5	17.8
Putative uncharacterized protein	1049780	38.4	6.1	76.7	1	5	5	18.3
Putative uncharacterized protein	1111275	21.8	9.0	70.8	1	3	3	14.8
DUF427	1046408	28.6	7.1	37.7	1	4	4	13.3
Putative uncharacterized protein	1078081	31.5	7.2	22.7	1	2	2	11.9
Putative uncharacterized protein	161080	26.2	5.5	13.0	1	3	3	18.9
Putative uncharacterized protein	1113075	53.5	5.6	11.2	1	2	2	4.8
